# Molecular Profiling and Targeted Therapeutic Strategies in Breast Cancer: Clinical Integration of HER2, CDK4/6, and PI3K Inhibition with Trastuzumab, Abemaciclib and Alpelisib

**DOI:** 10.3390/jcm15103715

**Published:** 2026-05-12

**Authors:** Piotr Kawczak, Tomasz Bączek

**Affiliations:** 1 Department of Pharmaceutical Chemistry, Faculty of Pharmacy, Medical University of Gdańsk, 80-416 Gdansk, Poland; tomasz.baczek@gumed.edu.pl; 2 Department of Nursing and Medical Rescue, Institute of Health Sciences, Pomeranian University in Słupsk, 76-200 Slupsk, Poland

**Keywords:** breast cancer, trastuzumab, abemaciclib, alpelisib, HER2, CDK4/6, PI3K, targeted therapy, precision oncology, molecular pathways

## Abstract

Advances in molecular oncology have reshaped the management of breast cancer through the development of pathway-specific targeted therapies. In particular, inhibition of HER2, CDK4/6, and PI3K signaling has yielded substantial clinical benefits in molecularly defined patient populations. This review provides an integrated analysis of three representative agents—trastuzumab, abemaciclib, and alpelisib—highlighting their distinct mechanisms of action, clinical efficacy, and translational relevance in breast cancer, with contextual insights into gynecologic oncology. Evidence from pivotal clinical trials and emerging translational studies demonstrates that trastuzumab remains a cornerstone of HER2-positive breast cancer treatment, while also showing activity in HER2-amplified gynecologic malignancies. Abemaciclib, a selective CDK4/6 inhibitor, has significantly improved outcomes in hormone receptor–positive breast cancer and is being actively explored in tumors characterized by cell cycle dysregulation, including endometrial and ovarian cancers. Alpelisib, targeting the PI3Kα isoform, provides meaningful benefit in PIK3CA-mutated breast cancer and represents a promising strategy in gynecologic tumors with aberrant PI3K/AKT/mTOR pathway activation. Collectively, these agents exemplify precision oncology approaches that align therapeutic strategies with tumor biology. Their integration into biomarker-driven, multimodal treatment frameworks underscores a paradigm shift toward personalized cancer care across breast and gynecologic malignancies. Particular emphasis is placed on the translation of molecular diagnostics into clinical decision-making, including patient selection, resistance mechanisms, and sequencing strategies within evolving precision oncology frameworks. Ongoing clinical and translational research will be critical to refine combination strategies, overcome resistance mechanisms, and identify predictive biomarkers to further optimize patient outcomes.

## 1. Introduction

Breast cancer remains the most frequently diagnosed malignancy among women worldwide and continues to represent one of the leading causes of cancer-related mortality [1]. Despite advances in screening, early detection, and systemic treatment, the global burden of this disease remains substantial, highlighting the ongoing need for biologically informed therapeutic strategies and improved precision medicine approaches [2,3,4,5]. Over the past two decades, major advances in molecular biology and genomic profiling have fundamentally reshaped the understanding of breast cancer. What was once considered a relatively uniform disease defined primarily by histopathological features is now recognized as a heterogeneous group of malignancies characterized by distinct molecular signatures, signaling pathways, and clinical behaviors [6,7,8,9,10,11]. This growing knowledge has provided critical insights into the mechanisms underlying tumor initiation, progression, and therapeutic resistance, ultimately facilitating the development of targeted therapies designed to selectively inhibit oncogenic signaling networks [12,13,14,15,16,17].

Current molecular classification systems stratify breast cancer into clinically and biologically relevant subtypes, most commonly including hormone receptor–positive (HR+)/human epidermal growth factor receptor 2 (HER2)–negative tumors, HER2-positive disease, and triple-negative breast cancer (TNBC). These entities exhibit distinct molecular profiles, therapeutic vulnerabilities, and clinical outcomes [6,7,8]. Within HR-positive disease, Luminal B tumors represent a biologically heterogeneous subgroup that includes both HER2-negative cancers with a high proliferative index (e.g., elevated Ki-67) and HER2-positive tumors co-expressing hormone receptors, reflecting differences in prognosis and treatment sensitivity [18,19]. HR+ tumors represent the most common subtype and are largely driven by estrogen receptor–mediated signaling pathways. HER2-positive tumors are defined by amplification or overexpression of the ERBB2 gene and are associated with increased proliferative capacity and historically poorer outcomes prior to the introduction of targeted therapy. In contrast, TNBC is defined by the absence of estrogen receptor (ER), progesterone receptor (PR), and HER2 expression and is characterized by pronounced genomic instability, an elevated mutational burden, and substantial intratumoral heterogeneity [20,21]. These features underlie its aggressive clinical behavior, higher risk of early recurrence, and limited availability of durable targeted treatment options, while also providing a biological rationale for emerging therapeutic approaches, including immune checkpoint inhibition and DNA damage response–targeted therapies [22,23,24]. More recently, additional biological subsets such as HER2-low breast cancer have been recognized, reflecting the increasing complexity of breast cancer classification and its therapeutic implications. HER2-low tumors are defined by low levels of HER2 expression, typically corresponding to an immunohistochemistry (IHC) score of 1+ or 2+ in the absence of HER2 gene amplification by in situ hybridization (ISH). This subgroup has gained clinical relevance with the advent of novel HER2-targeted antibody–drug conjugates, which have demonstrated meaningful activity in this previously unaddressed population [25,26,27].

Among the molecular pathways that play central roles in breast cancer biology, HER2, cyclin-dependent kinases 4 and 6 (CDK4/6), and phosphatidylinositol 3-kinase (PI3K) signaling cascades have emerged as particularly important therapeutic targets. These pathways regulate essential cellular processes, including proliferation, survival, metabolic signaling, and cell cycle progression. Dysregulation of these signaling networks can drive tumorigenesis and contribute to disease progression and resistance to therapy [28,29,30,31]. Importantly, alterations in these pathways define clinically actionable molecular subsets of breast cancer and have therefore become the focus of targeted therapeutic development [32,33,34,35].

Despite the substantial clinical benefit associated with cyclin-dependent kinase 4 and 6 (CDK4/6) inhibitors, such as abemaciclib, both intrinsic and acquired resistance remain major clinical challenges. Mechanisms of resistance include loss of retinoblastoma protein (RB1) function, which disrupts cell cycle control, as well as activation of compensatory signaling pathways, including fibroblast growth factor receptor (FGFR) signaling and PI3K/AKT/mTOR axis, enabling continued tumor proliferation despite CDK4/6 inhibition [36,37,38,39]. These molecular alterations highlight the dynamic nature of tumor evolution and support the emerging role of liquid biopsy approaches, including circulating tumor DNA (ctDNA), for real-time monitoring of resistance mechanisms and treatment adaptation [40,41].

HER2 overexpression or gene amplification occurs in approximately 15–20% of breast cancers and is associated with aggressive tumor biology and unfavorable prognosis in the absence of targeted intervention [42,43,44]. The clinical landscape of HER2-positive breast cancer changed dramatically following the introduction of trastuzumab, a humanized monoclonal antibody directed against the extracellular domain of the HER2 receptor. By inhibiting receptor signaling, preventing receptor dimerization, and stimulating antibody-dependent cellular cytotoxicity (ADCC), trastuzumab significantly improved survival outcomes in both early-stage and metastatic disease [44,45,46,47,48]. Subsequent therapeutic innovations, including antibody–drug conjugates, dual HER2 blockade strategies, and tyrosine kinase inhibitors, have further expanded the treatment options available for this subtype. Together, these developments illustrate the successful translation of molecular discoveries into clinically effective therapies [49,50,51,52,53].

Hormone receptor–positive breast cancers, which account for nearly 70% of diagnosed cases, are typically driven by estrogen-dependent signaling pathways and are therefore commonly treated with endocrine therapies such as aromatase inhibitors or selective ER modulators [54,55,56]. Nevertheless, endocrine resistance frequently develops during disease progression. One of the key mechanisms underlying this resistance involves dysregulation of the cyclin D–CDK4/6–retinoblastoma (Rb) signaling axis, which promotes uncontrolled cell cycle progression and sustained tumor proliferation. The development of selective CDK4/6 inhibitors, including abemaciclib, palbociclib, and ribociclib, has therefore represented a major therapeutic advance in HR+ breast cancer management [57,58,59,60]. By blocking CDK4/6 activity and restoring cell cycle control, these agents enhance the efficacy of endocrine therapy and significantly prolong progression-free survival (PFS) across multiple clinical trials [61,62,63].

The PI3K/AKT/mTOR signaling pathway represents another critical regulatory network involved in breast cancer pathogenesis. Activating mutations in the PIK3CA gene and other upstream alterations frequently lead to constitutive activation of this pathway, promoting tumor growth, metabolic adaptation, and resistance to endocrine or HER2-directed therapies [64,65,66]. As a result, pharmacological inhibition of PI3K signaling has emerged as an important therapeutic strategy [67,68,69]. Alpelisib, a selective inhibitor of the PI3Kα isoform, has demonstrated significant clinical benefit in patients with PIK3CA-mutated HR+/HER2− advanced breast cancer when administered in combination with endocrine therapy [70]. These findings highlight the growing importance of genomic testing and biomarker-guided treatment selection in modern breast cancer management [35,71].

Despite the substantial progress achieved through targeted therapies, several challenges continue to limit long-term treatment success. Both intrinsic and acquired resistance frequently arise during therapy, often driven by compensatory signaling pathways, tumor microenvironment interactions, or clonal evolution within the tumor cell population [72,73,74]. Furthermore, the complex interplay between tumor cells, immune components, and stromal elements within the tumor microenvironment can influence therapeutic response and contribute to disease progression. Ongoing research efforts are therefore focused on identifying predictive biomarkers, optimizing treatment sequencing, and developing rational combination strategies capable of overcoming resistance mechanisms and improving durable clinical responses [73,75]. Beyond conventional HER2-targeted strategies, emerging approaches include combination therapies integrating trastuzumab with immune checkpoint inhibitors, aiming to enhance ADCC and promote antitumor immune responses [76,77]. In parallel, antibody–drug conjugates (ADCs), such as trastuzumab deruxtecan, represent a major therapeutic advancement by coupling HER2 targeting with intracellular delivery of cytotoxic payloads, thereby extending clinical benefit even to tumors with low HER2 expression [78,79].

This narrative review explores the interface between molecular biology and clinical application in breast cancer therapy, with particular emphasis on the HER2, CDK4/6, and PI3K signaling pathways. The review summarizes the mechanisms of action, key clinical trial data, and therapeutic positioning of trastuzumab, abemaciclib, and alpelisib while also discussing emerging resistance mechanisms and evolving combination strategies. Special attention is given to precision oncology approaches and biomarker-driven treatment selection, which continue to reshape the therapeutic landscape of breast cancer management.

A structured literature search was conducted using the PubMed and Scopus databases to identify relevant English-language publications published between 2006 and March 2026. The starting point was selected to encompass early translational and clinical investigations of targeted therapies that preceded or coincided with their subsequent regulatory approvals. Search terms included combinations of “breast cancer,” “trastuzumab,” “abemaciclib,” “alpelisib,” “HER2,” “CDK4/6,” “PI3K,” “immune checkpoint inhibitors,” “targeted therapy,” and “combination therapy.” Eligible studies comprised phase II and III prospective trials, randomized controlled trials, registration-directed studies, meta-analyses, and clinically informative prospective cohort studies evaluating these agents. Priority was given to randomized trials and pivotal studies supporting regulatory approvals, while large real-world cohorts and retrospective analyses were considered when they provided meaningful insights into treatment outcomes, safety, or therapeutic sequencing.

The schematic figures included in this manuscript were created by the authors using graphical software that may incorporate artificial intelligence–assisted functionalities to support visualization. All scientific content, interpretation, and final figure design were critically reviewed and approved by the authors, who take full responsibility for the accuracy and originality of the material.

**Figure 1:**
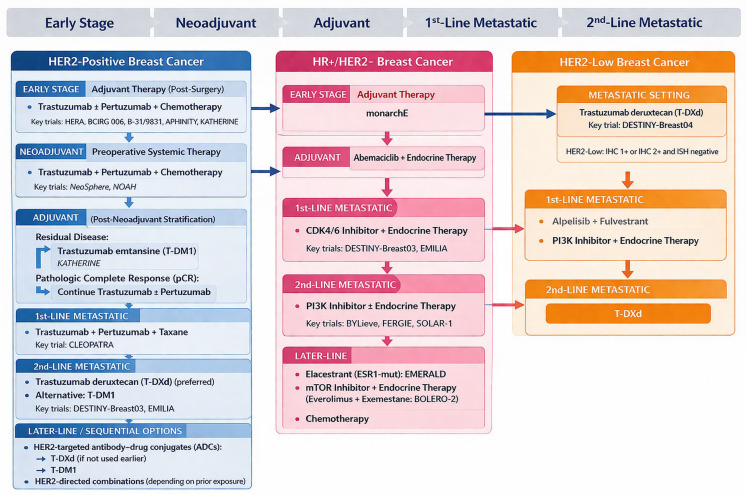
Schematic overview of treatment selection breast cancer, integrating pivotal clinical trial data by biomarker status and line of therapy. The scheme represents an original, interpretative synthesis of current evidence aligned with key NCCN and ESMO principles, including biomarker-driven treatment selection and therapy sequencing. It constitutes an original graphical representation and does not reproduce or replicate any specific published guideline or source. All abbreviations employed are defined in the text in the Abbreviations section.

Figure 1 provides a schematic overview of breast cancer treatment selection, incorporating key clinical trial evidence stratified by biomarker status and line of therapy.

## 2. Trastuzumab

**Figure 2:**
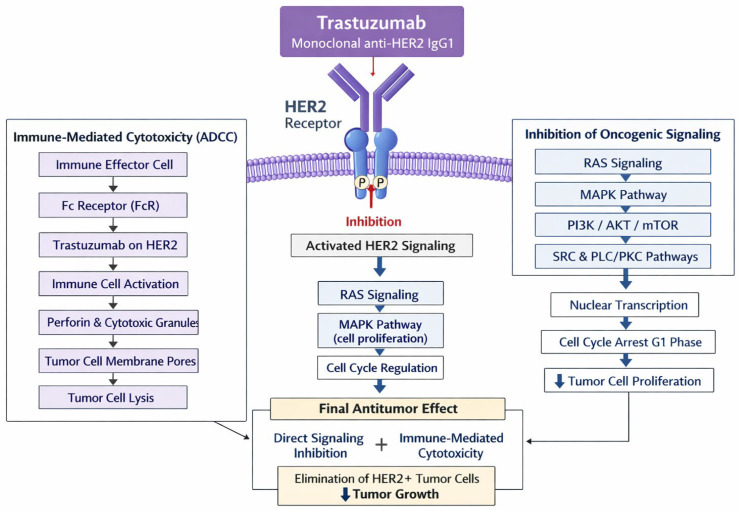
Trastuzumab mechanism of action. Original schematic illustration created by the authors based on the mechanism described in [90]. HER2 is a transmembrane receptor of the HER family that, when overexpressed in certain breast cancers, drives tumor growth through activation of signaling pathways such as PI3K/Akt and MAPK, while the humanized monoclonal antibody trastuzumab—derived from the murine antibody 4D5—targets the extracellular domain of HER2 to inhibit receptor signaling, block receptor cleavage and dimerization, induce cell-cycle arrest, reduce angiogenesis, and promote ADCC against HER2-positive tumor cells. All abbreviations employed are defined in the text in the Abbreviations section.

Trastuzumab is a humanized monoclonal antibody directed against the extracellular domain of HER2, representing a prototypical targeted biologic that has fundamentally reshaped the natural history of HER2-positive breast cancer [80,81,82,83,84]. As an IgG1 antibody, it exerts antitumor activity through multiple complementary mechanisms, including inhibition of ligand-independent HER2 signaling, prevention of receptor dimerization, promotion of receptor internalization and degradation, and engagement of immune-mediated cytotoxicity via ADCC [80,81,85]. Beyond simple receptor blockade, trastuzumab modulates downstream pathways such as PI3K/AKT and MAPK, thereby reducing proliferation and enhancing apoptosis [81,82,86]. These multimodal effects distinguish it from small-molecule tyrosine kinase inhibitors and partially explain its durable clinical efficacy despite the emergence of resistance mechanisms [87,88,89]. Figure 2 illustrates the mechanism of action of trastuzumab.

The clinical development of trastuzumab marked a turning point in precision oncology. The first pivotal evidence of benefit emerged from a landmark randomized trial in metastatic HER2-overexpressing breast cancer, which demonstrated that the addition of trastuzumab to chemotherapy significantly improved response rates, PFS, and overall survival (OS), establishing HER2 as a predictive biomarker and validating targeted therapy as a therapeutic paradigm [45,91]. Importantly, this study also highlighted early safety concerns, particularly cardiotoxicity when combined with anthracyclines, an issue that would shape subsequent trial designs and clinical practice [45,92]. A subsequent study (M77001) confirmed the benefit of combining trastuzumab with docetaxel in the first-line metastatic setting, reinforcing the concept that HER2 blockade synergizes with cytotoxic therapy [93].

The translation of trastuzumab into early-stage disease was rapidly pursued and remains one of the most successful examples of adjuvant targeted therapy. The HERA trial established one year of adjuvant trastuzumab following chemotherapy as a standard of care, demonstrating significant improvements in disease-free survival (DFS) and OS [46,94]. Notably, while longer durations of therapy were explored, no additional survival benefit was observed beyond one year, underscoring the importance of balancing efficacy with toxicity and cost [95]. Parallel North American trials (NSABP B-31 and NCCTG N9831) showed that concurrent administration of trastuzumab with taxane-based chemotherapy resulted in marked improvements in DFS and OS compared with chemotherapy alone [96]. These trials differed in sequencing and design, yet converged on similar conclusions, strengthening the robustness of the evidence. However, cross-trial comparisons should be interpreted cautiously due to differences in patient populations and chemotherapy backbones.

The BCIRG 006 trial further refined treatment strategies by comparing anthracycline-containing and non-anthracycline regimens. While efficacy outcomes were broadly comparable, the non-anthracycline regimen (docetaxel, carboplatin, and trastuzumab) demonstrated a more favorable cardiac safety profile, providing a critical alternative for patients at increased cardiovascular risk [97]. This finding has had lasting clinical implications, particularly in tailoring therapy for older patients or those with comorbidities. Nevertheless, subtle differences in long-term outcomes and patient selection criteria suggest that regimen choice should remain individualized rather than universally standardized.

In the neoadjuvant setting, trastuzumab has enabled significant improvements in pathological complete response (pCR), a surrogate endpoint associated with long-term outcomes in HER2-positive disease [98]. The NOAH trial demonstrated that the addition of trastuzumab to chemotherapy significantly increased pCR rates and event-free survival in locally advanced and inflammatory breast cancer [99]. The NeoSphere trial extended these findings by introducing dual HER2 blockade with trastuzumab and pertuzumab, showing higher pCR rates compared with single-agent HER2 targeting [89,94,100]. While pCR is an attractive early endpoint, its correlation with long-term survival varies across subtypes and treatment contexts, and caution is warranted when extrapolating short-term results to definitive clinical benefit.

The adjuvant landscape has further evolved with the incorporation of dual HER2 blockade. The APHINITY trial demonstrated that adding pertuzumab to trastuzumab and chemotherapy modestly improved invasive DFS, with the greatest benefit observed in node-positive patients [101]. The relatively small absolute benefit, however, raises important questions regarding cost-effectiveness and patient selection, emphasizing the need for biomarker-driven refinement. Notably, long-term follow-up of APHINITY has reinforced the durability of benefit, particularly in high-risk populations, while highlighting the generally manageable safety profile of dual HER2-targeted therapy [101,102]. In contrast, the KATHERINE trial addressed patients with residual disease after neoadjuvant therapy, showing that switching from trastuzumab to trastuzumab emtansine (T-DM1) significantly improved invasive DFS [103,104,105]. This study exemplifies response-adapted therapy and highlights the limitations of trastuzumab monotherapy in high-risk, treatment-resistant populations, supporting the use of residual disease as a predictive biomarker for escalation.

Neoadjuvant therapy strategies have also evolved, as highlighted by the GeparSepto-GBG 69 trial, which compared nab-paclitaxel with solvent-based paclitaxel in combination with standard neoadjuvant regimens for early breast cancer [106]. The study demonstrated higher pathological complete response (pCR) rates with nab-paclitaxel, particularly in aggressive subtypes such as HER2-positive and triple-negative tumors, underscoring the importance of optimizing chemotherapy backbones to enhance HER2-targeted therapy efficacy [106]. These findings have informed subsequent neoadjuvant strategies, supporting individualized treatment selection based on tumor biology and anticipated response [107,108].

In metastatic disease, trastuzumab remains a foundational component of first-line therapy. The CLEOPATRA trial established the combination of trastuzumab, pertuzumab, and docetaxel as a standard of care, demonstrating substantial improvements in both PFS and OS [84,109,110]. The magnitude and durability of benefit observed in this trial are notable, although patient selection (e.g., exclusion of prior anti-HER2 therapy in the metastatic setting) may limit generalizability to contemporary populations increasingly exposed to trastuzumab earlier in the disease course. Subsequent lines of therapy have been transformed by antibody–drug conjugates such as T-DM1 (EMILIA trial) and trastuzumab deruxtecan (DESTINY-Breast03), both of which build upon the trastuzumab backbone to deliver cytotoxic payloads directly to HER2-expressing cells [78,111,112]. These agents have demonstrated superior efficacy compared with earlier standards, although differences in toxicity profiles, particularly interstitial lung disease with trastuzumab deruxtecan, necessitate careful monitoring [113,114,115,116]. Moreover, evolving evidence supports the sequential use of these agents based on prior HER2-directed therapies and patient tolerability, further personalizing metastatic treatment [26,114,117].

A particularly significant development is the expansion of HER2-targeted therapy into the HER2-low population, as demonstrated in the DESTINY-Breast04 trial [105,113,115]. This study showed that trastuzumab deruxtecan significantly improved PFS and OS compared with chemotherapy in patients with low HER2 expression (IHC 1+ or 2+/ISH-negative), effectively redefining HER2 as a spectrum rather than a binary biomarker [26,113,114]. While trastuzumab itself has limited activity in this subgroup, its role as a targeting moiety in antibody–drug conjugates underscores its continued relevance [26,112]. These findings challenge traditional classification systems and highlight the dynamic interplay between diagnostic criteria, therapeutic innovation, and the need for precise biomarker testing [43]. Ongoing studies are now evaluating combination strategies in HER2-low disease, including integration with endocrine therapy and immunotherapy [118,119,120].

Despite its transformative impact, resistance to trastuzumab remains a major clinical challenge. Mechanisms include alterations in the HER2 receptor (e.g., truncated forms such as p95HER2), activation of alternative signaling pathways (e.g., PI3K mutations, PTEN loss), and immune evasion [27,83,86,121]. These resistance pathways have driven the development of combination strategies, including dual HER2 blockade, integration with CDK4/6 inhibitors, and PI3K pathway targeting [27,102]. While preclinical rationale is strong, clinical results have been heterogeneous, reflecting the complexity of tumor biology and the need for more precise biomarker-driven approaches. Novel strategies, including antibody–drug conjugates and bispecific antibodies, are being investigated to overcome these resistance mechanisms, providing hope for patients with refractory disease [105,112,122].

The safety profile of trastuzumab is generally favorable compared with conventional chemotherapy, but cardiotoxicity remains the most clinically significant adverse effect [92,123]. Unlike anthracycline-induced cardiomyopathy, trastuzumab-associated cardiac dysfunction is often reversible and not dose-dependent, yet it necessitates regular cardiac monitoring [92]. Infusion-related reactions are relatively common, particularly during the first administration, but are typically manageable with premedication and supportive care [27,103,124,125]. Long-term tolerability has facilitated extended use in both adjuvant and metastatic settings, although cumulative toxicity and patient quality of life must be considered in treatment planning. Importantly, subcutaneous formulations of trastuzumab have demonstrated equivalent efficacy with improved patient convenience, which may enhance adherence in real-world practice [103,124,125].

In contemporary practice, trastuzumab serves not only as a therapeutic agent but also as a platform for drug development, forming the backbone of multiple combination regimens and antibody–drug conjugates [36,43,83,104]. Its integration with other targeted therapies, including CDK4/6 and PI3K inhibitors, reflects an evolving paradigm in which HER2 signaling is considered within a broader network of oncogenic pathways [102]. However, variability in trial design, patient populations, and endpoints complicates cross-study comparisons and highlights the importance of critical interpretation when translating evidence into clinical decision-making. Adaptive trial designs and biomarker-enriched cohorts are increasingly leveraged to optimize both efficacy and safety outcomes.

Overall, trastuzumab exemplifies the success of biomarker-driven therapy in oncology, yet its optimal use continues to evolve [43,126,127,128]. While early trials established its efficacy across disease stages, more recent studies have focused on treatment de-escalation, escalation in high-risk populations, and expansion into new biomarker-defined subgroups. The challenge moving forward lies in refining patient selection, overcoming resistance, and integrating trastuzumab-based strategies within increasingly complex therapeutic landscapes, ensuring that both efficacy and tolerability are maintained across diverse patient populations [83,84,85].

Table 1 summarizes treatment-emergent adverse events (TEAEs) and their management strategies for trastuzumab, while Table 2 outlines the major pivotal clinical trials of trastuzumab in breast cancer.

## 3. Abemaciclib

Abemaciclib is an orally bioavailable, small-molecule inhibitor of CDK4/6, a class of targeted agents that has fundamentally reshaped the therapeutic landscape of HR+, HER2− breast cancer [132,133]. Unlike conventional cytotoxic therapies, CDK4/6 inhibitors exploit a central vulnerability of luminal breast cancer biology—dysregulated cell-cycle progression driven by cyclin D–CDK4/6–Rb signaling [134,135,136]. Abemaciclib demonstrates relatively greater selectivity for CDK4 over CDK6 and supports continuous dosing, features that likely contribute to its distinct pharmacodynamic and toxicity profile within this drug class [137,138].

**Figure 3:**
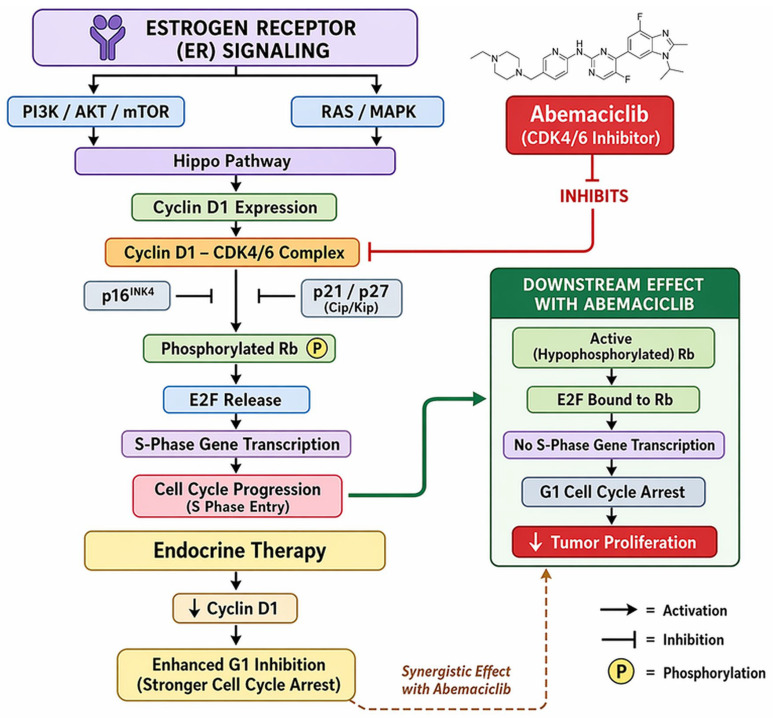
Abemaciclib mechanism of action. Original schematic illustration created by the authors based on the mechanism described in [145]. Abemaciclib, a selective CDK4/6 inhibitor, blocks phosphorylation of the retinoblastoma (Rb) protein, thereby preventing cell-cycle progression from the G1 to S phase and inhibiting tumor cell proliferation. All abbreviations employed are defined in the text in the Abbreviations section.

Mechanistically, abemaciclib inhibits phosphorylation of the Rb protein, thereby enforcing G1 cell-cycle arrest and suppressing proliferation in Rb-competent tumor cells [134,135,136]. Beyond canonical cell-cycle inhibition, preclinical studies suggest broader biological effects, including modulation of transcriptional programs and the tumor microenvironment, with evidence of enhanced antigen presentation and potential synergy with endocrine therapy [36,139]. Translational analyses from the neoMONARCH neoadjuvant study further demonstrated that abemaciclib combined with anastrozole induced profound suppression of proliferation markers such as Ki-67 and enhanced immune-related gene expression signatures, supporting the hypothesis that CDK4/6 inhibition may exert immunomodulatory effects beyond cell-cycle blockade [140]. Its continuous dosing schedule—facilitated by a comparatively lower incidence of severe neutropenia—enables sustained target inhibition, which may be particularly relevant in tumors with high proliferative activity or adaptive resistance mechanisms [141,142,143]. Emerging translational studies further suggest that abemaciclib may influence tumor biology through broader genomic and epigenetic mechanisms, including alterations in circulating chromatin fragments and transcriptional regulation that could potentially serve as biomarkers of therapeutic response [144]. Figure 3 depicts the mechanism of action of abemaciclib.

The clinical development of abemaciclib broadly parallels that of other CDK4/6 inhibitors but introduces important distinctions in therapeutic positioning. Early-phase studies demonstrated single-agent activity in heavily pretreated metastatic breast cancer, a feature less pronounced with other agents in this class [137]. The phase II MONARCH 1 trial confirmed this activity, reporting objective responses in refractory HR+/HER2− metastatic disease [141,146]. However, the single-arm design and heavily pretreated population limit cross-trial comparisons and underscore the need for contextual interpretation relative to evolving standards of care [142,147].

Subsequent randomized trials established abemaciclib as a cornerstone of combination therapy across both endocrine-sensitive and endocrine-resistant settings. In MONARCH 2, abemaciclib plus fulvestrant significantly improved PFS and OS compared with fulvestrant alone in patients progressing on prior endocrine therapy [58]. The survival advantage observed in this trial was later confirmed in mature analyses demonstrating a clinically meaningful improvement in median OS [148], reinforcing the therapeutic relevance of CDK4/6 inhibition after endocrine resistance. Notably, the demonstration of an OS benefit is clinically meaningful in this context, although interpretation should account for heterogeneity in prior treatments and patient populations [142]. MONARCH 3 evaluated abemaciclib in combination with nonsteroidal aromatase inhibitors as initial therapy for advanced disease, demonstrating substantial improvements in PFS [149]. Updated long-term analyses have now demonstrated that the combination also confers a sustained OS advantage, reinforcing the durability of benefit associated with early CDK4/6 inhibition [150]. While OS data remain influenced by subsequent lines of therapy, the magnitude and consistency of PFS benefit support early integration of CDK4/6 inhibition [142,147].

Several considerations emerge from critical appraisal of these trials. Differences in inclusion criteria—including prior endocrine exposure, disease burden, and menopausal status—limit direct comparisons across studies and agents [142,151]. Moreover, reliance on PFS as a primary endpoint, while clinically relevant, may not fully capture long-term outcomes such as durability of response or quality of life [147]. Additionally, crossover and post-progression therapies complicate OS interpretation [142]. Despite these limitations, the consistency of benefit across trials reinforces the robustness of CDK4/6 inhibition as a therapeutic paradigm [132,139]. Real-world analyses comparing abemaciclib, palbociclib, and ribociclib have similarly suggested comparable clinical effectiveness across the class while emphasizing differences in toxicity profiles and dosing strategies that may influence treatment selection in clinical practice [152,153]. Recent observational data from patient support programs further indicate high treatment satisfaction and acceptable tolerability in routine practice, highlighting the importance of supportive care and patient education in maintaining adherence to continuous therapy [154].

The role of abemaciclib has expanded into early breast cancer. The MONARCH-E trial evaluated adjuvant abemaciclib in combination with endocrine therapy in patients with high-risk, node-positive HR+/HER2− disease, demonstrating a significant improvement in invasive disease-free survival (IDFS) [155,156]. Safety analyses and patient-reported outcomes from this trial confirmed the manageable toxicity profile and acceptable quality-of-life impact associated with prolonged therapy [157]. These findings supported regulatory approval in selected high-risk populations [158]. However, several caveats warrant consideration. Definitions of “high risk” vary across trials, and follow-up duration remains relatively limited for assessing long-term survival outcomes [158,159]. Furthermore, optimal treatment duration and patient selection criteria continue to evolve, particularly with increasing use of genomic risk stratification tools [160,161,162]. Recent analyses integrating genomic recurrence scores and endocrine responsiveness suggest that molecular stratification may further refine identification of patients most likely to benefit from adjuvant CDK4/6 inhibition [159], and updated treatment algorithms increasingly incorporate such approaches in routine clinical decision-making [163,164]. Parallel surgical oncology research has also explored how evolving systemic therapy indications may influence locoregional management decisions, including axillary surgery strategies in patients potentially eligible for adjuvant abemaciclib [165].

Combination strategies involving abemaciclib are increasingly informed by resistance biology and pathway crosstalk. Insights from PI3K pathway inhibition trials, including SOLAR-1 and BYLieve, have established the clinical relevance of PIK3CA mutations and the role of alpelisib in endocrine-resistant disease, particularly following CDK4/6 inhibitor exposure [70,166,167]. Notably, the BYLieve study provides prospective evidence that PI3K inhibition retains activity after progression on CDK4/6 inhibitors [153]. These findings suggest that resistance to CDK4/6 inhibition does not preclude responsiveness to downstream pathway targeting [168,169]. Nonetheless, differences in trial design and patient selection highlight the need for integrated, biomarker-driven treatment strategies [70,161]. More recently, the phase III postMONARCH trial demonstrated that abemaciclib combined with fulvestrant retains clinical activity in patients previously treated with a CDK4/6 inhibitor, suggesting that continued targeting of the cyclin D–CDK4/6–Rb axis may remain therapeutically relevant in selected patients [170].

Additional clinical investigations have explored broader combinatorial strategies. The monarcHER phase II trial demonstrated that abemaciclib combined with trastuzumab, with or without fulvestrant, provided a chemotherapy-free treatment option for patients with HR-positive, HER2-positive advanced breast cancer, highlighting the potential role of CDK4/6 inhibition in HER2-driven disease contexts [171]. Emerging endocrine combinations are also being evaluated; for example, analyses of phase III trials involving the oral selective ER degrader imlunestrant suggest that dual targeting of ER signaling and CDK4/6 may further improve outcomes in endocrine-resistant disease [172].

Earlier PI3K inhibitor trials, including SANDPIPER, BELLE-2, and BELLE-3, further illustrate both the therapeutic potential and limitations of this pathway [168,173,174]. Although improvements in PFS were observed, toxicity—particularly with pan-PI3K inhibitors—limited widespread clinical adoption [173,174,175]. In contrast, the more favorable therapeutic index of abemaciclib has supported its broader clinical use, despite gastrointestinal toxicity remaining a key management consideration [141]. Emerging therapeutic strategies are also exploring combinations with next-generation endocrine agents. For example, the phase III EMBER-3 trial evaluating the oral selective ER degrader imlunestrant with or without abemaciclib demonstrated improved outcomes with the combination strategy, highlighting the potential of dual endocrine and cell-cycle targeting in advanced HR+/HER2− disease [176,177].

Adverse events associated with abemaciclib are generally predictable and managea-ble but exhibit clinically relevant differences compared with other CDK4/6 inhibitors. Di-arrhea is the most common toxicity, typically occurring early and requiring proactive management with antidiarrheal agents and dose modifications [45,141]. Neutropenia is less frequent and less severe, reflecting differential kinase selectivity [142]. Hepatotoxicity and venous thromboembolism have also been reported and warrant routine monitoring [156]. The continuous dosing schedule further necessitates vigilance for cumulative toxici-ties, particularly in the adjuvant setting [156,159]. Recent pharmacovigilance and me-ta-analytic studies have highlighted the association between abemaciclib and thromboembolic events, emphasizing the importance of individualized risk assessment [178,179]. Post-marketing safety analyses have also identified rare cardiovascular and arrhythmic events associated with CDK4/6 inhibitors, underscoring the importance of ongoing moni-toring in clinical practice [180,181].

From a translational perspective, resistance to abemaciclib arises through diverse mechanisms, including loss of Rb function, activation of cyclin E–CDK2 signaling, and engagement of alternative proliferative pathways such as PI3K/AKT/mTOR [134,136,175,182]. Emerging evidence also implicates additional genomic alterations, in-cluding aberrant FGFR signaling, in mediating resistance and therapeutic escape [37]. These insights have prompted investigation of rational combination strategies incorpo-rating PI3K inhibitors, mTOR inhibitors, and next-generation endocrine therapies [183,184]. However, clinical implementation remains constrained by overlapping toxici-ties and the need for validated predictive biomarkers [36,159].

The positioning of abemaciclib within contemporary treatment algorithms reflects a broader shift toward precision oncology. Biomarker-driven approaches—such as PIK3CA mutation testing—are increasingly integrated into clinical decision-making, although ro-bust predictive biomarkers for CDK4/6 inhibitor response remain limited [70,161]. Ongo-ing efforts to define genomic and transcriptomic predictors of response may refine patient selection and optimize therapeutic sequencing [162,185,186]. Real-world surveys and ob-servational studies further indicate broad adoption of CDK4/6 inhibitors in routine on-cology practice, reflecting confidence in their clinical efficacy and manageable safety pro-files [187,188].

In summary, abemaciclib represents a central component of modern breast cancer management, with demonstrated efficacy across multiple disease settings and a distinct pharmacologic profile among CDK4/6 inhibitors [132,133,139]. Its clinical development underscores both the strengths and limitations of current evidence, highlighting the im-portance of careful trial interpretation and biomarker integration [142,147]. As treatment paradigms continue to evolve, the future role of abemaciclib will likely be shaped by ad-vances in resistance biology, rational combination strategies, and increasingly personal-ized therapeutic approaches [38,183,186,189]. Continued integration of translational re-search, real-world data, and emerging endocrine therapies will further refine the optimal positioning of CDK4/6 inhibition in precision oncology [190].

Table 3 summarizes TEAEs and their management strategies for abemaciclib, while Table 4 outlines the major pivotal clinical trials of abemaciclib in breast cancer.

## 4. Alpelisib

Alpelisib is an orally bioavailable, selective inhibitor of the phosphatidylinositol-3-kinase (PI3K) catalytic subunit p110α, representing a paradigm shift toward biomarker-driven therapy in HR+, HER2− breast cancer [70,167]. Unlike earlier pan-PI3K inhibitors, alpelisib was rationally developed to target tumors harboring activating mutations in the PIK3CA gene, which occur in approximately 30–40% of HR+ breast cancers and are associated with endocrine resistance and disease progression [196,197,198]. This molecular selectivity aims to improve the therapeutic index by limiting off-target toxicity while maintaining pathway inhibition, although in practice class-related adverse effects remain clinically significant [199,200,201].

**Figure 4:**
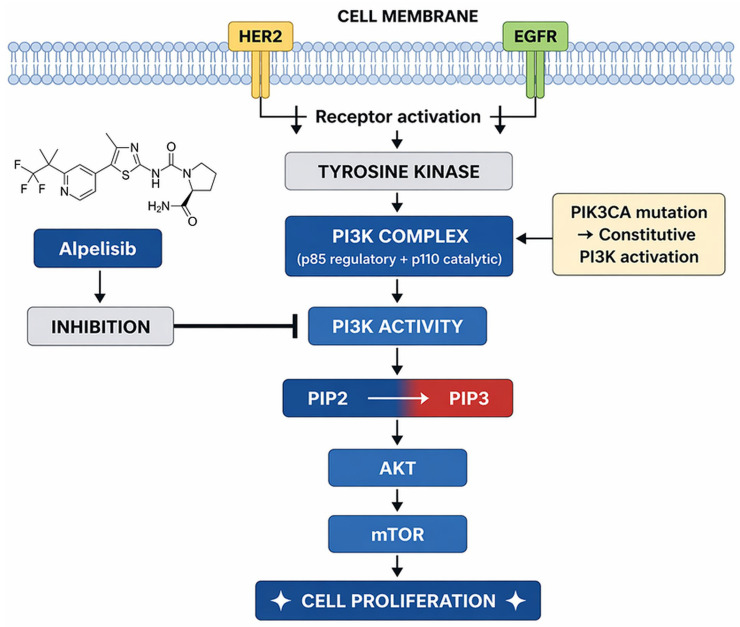
Alpelisib mechanism of action. Original schematic illustration created by the authors based on the mechanism described in [211]. Alpelisib is an oral selective PI3Kα inhibitor that targets PIK3CA-mutated tumors, suppressing PI3K/Akt signaling and thereby reducing tumor cell proliferation and promoting cell-cycle arrest. All abbreviations em-ployed are defined in the text in the Abbreviations section.

Mechanistically, alpelisib inhibits the PI3K/AKT/mTOR signaling cascade by selectively targeting the p110α isoform, thereby reducing downstream AKT activation, impairing cell proliferation, and promoting apoptosis in PIK3CA-mutant tumor cells [202,203,204]. The PI3K pathway plays a central role in mediating resistance to endocrine therapy, particularly through ligand-independent ER activation and cross-talk with growth factor signaling pathways [205,206,207]. Consequently, the therapeutic rationale for alpelisib is closely linked to its use in combination with endocrine therapy, most commonly fulvestrant, to restore hormone sensitivity and delay disease progression [198,208,209]. Preclinical data further suggest that PI3K inhibition may modulate tumor metabolism and the immune microenvironment, although the clinical relevance of these effects remains incompletely defined [202,210]. Figure 4 illustrates the mechanism of action of alpelisib.

The clinical development of alpelisib culminated in the phase III SOLAR-1 trial, which established its role in PIK3CA-mutant HR+/HER2− advanced breast cancer [166,200,207]. In this randomized study, alpelisib in combination with fulvestrant significantly improved PFS compared with fulvestrant alone in patients with documented PIK3CA mutations who had progressed on prior endocrine therapy [166,201,212]. Importantly, the benefit was largely confined to the biomarker-selected population, underscoring the necessity of molecular testing for appropriate patient selection [70,166,203]. However, the trial has several limitations that warrant careful interpretation. Notably, only a minority of patients had received prior CDK4/6 inhibitors, reflecting the evolving standard of care at the time of study design [133,207]. This limits the direct applicability of SOLAR-1 results to contemporary treatment sequencing, where CDK4/6 inhibitors are routinely used in the first-line setting [213,214].

The phase II BYLieve trial sought to address this gap by evaluating alpelisib in patients with PIK3CA-mutant disease following progression on CDK4/6 inhibitors [166,215]. This non-randomized study demonstrated clinically meaningful activity of alpelisib combined with endocrine therapy (either fulvestrant or an aromatase inhibitor), supporting its use in later-line settings and providing prospective evidence for treatment sequencing in real-world practice [166,215,216,217]. Nevertheless, the absence of a control arm and heterogeneity in prior treatments limit definitive conclusions regarding comparative efficacy [218,219]. These constraints highlight a broader challenge in oncology drug development, where rapidly evolving standards of care can outpace trial design and complicate cross-study comparisons.

Contextualizing alpelisib within the broader landscape of PI3K inhibition is essential for understanding both its strengths and limitations. Earlier phase III trials, including SANDPIPER (taselisib) and BELLE-2/BELLE-3 (buparlisib), demonstrated that targeting the PI3K pathway could improve PFS in HR+/HER2− breast cancer, particularly in PIK3CA-altered tumors [173,174,175,207]. However, the clinical utility of these agents was limited by unfavorable toxicity profiles, including psychiatric, hepatic, and metabolic adverse events associated with pan-PI3K inhibition [173,174,175,201,220]. In contrast, alpelisib’s isoform selectivity represents an important pharmacologic refinement, although its toxicity profile—particularly hyperglycemia and rash—remains clinically significant and requires proactive management [221,222,223].

Adverse events associated with alpelisib are largely on-target effects related to PI3K pathway inhibition in normal tissues. Hyperglycemia is the most common and clinically relevant toxicity, resulting from impaired insulin signaling and increased hepatic gluconeogenesis [224,225,226]. This adverse effect often necessitates initiation of antihyperglycemic therapy and may lead to dose interruptions or discontinuation in a subset of patients [200,221]. Rash, diarrhea, and stomatitis are also frequently observed and may require supportive care measures or corticosteroid prophylaxis [223,224,225]. Importantly, the incidence and severity of these toxicities underscore the need for careful patient selection, baseline metabolic assessment, and multidisciplinary management, particularly in patients with pre-existing metabolic comorbidities [214,227].

Combination strategies involving alpelisib reflect ongoing efforts to overcome resistance and optimize therapeutic sequencing. The combination with fulvestrant remains the most extensively studied and clinically validated approach, as demonstrated in SOLAR-1 and BYLieve [166,215]. Additional studies are exploring combinations with other endocrine agents, CDK4/6 inhibitors, and novel targeted therapies, including emerging ER degraders and PROTAC-based approaches [228,229,230,231]. Moreover, the integration of alpelisib into treatment algorithms must consider prior therapies, resistance mechanisms, and patient-specific factors, including tolerability and comorbidity burden [213,214,227].

From a translational perspective, resistance to alpelisib can arise through multiple mechanisms, including activation of alternative signaling pathways (e.g., MAPK), loss of PTEN function, and adaptive feedback loops within the PI3K/AKT/mTOR axis [203,232,233]. These insights have prompted investigation of rational combination strategies targeting parallel or downstream pathways, although clinical validation remains limited [234,235,236,237]. Furthermore, the heterogeneity of PIK3CA mutations and their differential functional impact may influence therapeutic response, highlighting the need for more refined biomarker strategies beyond simple mutation status [197,238].

The clinical positioning of alpelisib illustrates the broader transition toward precision oncology in breast cancer. The requirement for PIK3CA mutation testing prior to therapy initiation represents a clear example of biomarker-driven treatment selection, aligning therapeutic intervention with tumor biology [70,167,203]. However, several challenges remain. The predictive value of PIK3CA mutations is not absolute, and a subset of patients derives limited benefit despite harboring the target alteration [207,239]. Additionally, the optimal sequencing of alpelisib relative to other targeted therapies, including CDK4/6 and mTOR inhibitors, remains an area of active investigation [213,217,240].

In summary, alpelisib represents a clinically meaningful advance in the management of HR+/HER2− breast cancer, providing a targeted therapeutic option for patients with PIK3CA-mutant disease [70,166]. Its development highlights both the promise and complexity of precision oncology, demonstrating the importance of biomarker selection, rational combination strategies, and careful toxicity management [167,201,227]. While its efficacy is well established in appropriately selected populations, limitations related to toxicity, trial design, and evolving treatment landscapes necessitate nuanced interpretation of the available evidence [133,218,219]. Ongoing research aimed at refining patient selection, overcoming resistance mechanisms, and optimizing treatment sequencing will ultimately determine the long-term role of alpelisib within increasingly individualized treatment strategies [37,204,207,241]. In parallel, computational drug discovery approaches, including virtual screening and molecular dynamics simulations, continue to support the identification of novel PI3Kα inhibitors with potential therapeutic relevance in breast cancer [242].

Table 5 summarizes TEAEs and their management strategies for alpelisib, while Table 6 outlines the major pivotal clinical trials of alpelisib in breast cancer.

## 5. Emerging Therapeutic Strategies and Translational Perspectives in Breast Cancer

Emerging therapeutic strategies in breast cancer are increasingly shaped by advances in molecular oncology, biomarker-guided therapeutic selection, and adaptive treatment sequencing. The transition from largely empirical cytotoxic approaches toward precision oncology reflects a deeper understanding of tumor heterogeneity, clonal evolution, and complex signaling network interactions, particularly within HER2, CDK4/6, and PI3K/AKT/mTOR pathways [246,247,248]. These molecular insights have enabled the development of targeted agents that improve outcomes in defined patient populations. Therapies such as trastuzumab, abemaciclib, and alpelisib illustrate how molecular stratification can translate into meaningful clinical benefit, while simultaneously highlighting the complexity of optimizing individualized treatment strategies in the context of dynamic resistance mechanisms and pathway crosstalk [5,15,249]. Recent translational studies continue to identify novel molecular scaffolds and signaling regulators capable of modulating cell-cycle progression and oncogenic transcriptional programs in breast cancer cells [250].

The current standard of care remains multimodal, integrating surgery, radiotherapy, and systemic therapy tailored to disease stage and molecular subtype [251,252]. Surgery and radiotherapy are foundational for early-stage disease, with breast-conserving surgery combined with radiotherapy achieving survival outcomes comparable to mastectomy while preserving quality of life [251,252]. Adjuvant radiotherapy significantly reduces local recurrence and breast cancer mortality, although potential long-term adverse effects, including cardiopulmonary toxicity, must be considered [246,253]. Modern clinical guidelines increasingly emphasize multidisciplinary treatment planning to optimize outcomes across diverse patient populations [60].

Endocrine therapy remains the backbone of treatment for HR+ breast cancer, the most common molecular subtype. Agents such as tamoxifen, aromatase inhibitors, and fulvestrant consistently improve disease-free and OS [254,255]. The addition of CDK4/6 inhibitors has fundamentally transformed the therapeutic landscape of HR+/HER2− advanced breast cancer, as demonstrated in trials including MONARCH, PALOMA, and MONALEESA [58,256,257]. Resistance to endocrine therapy and CDK4/6 inhibition can arise through genomic alterations such as ESR1 mutations, cyclin E amplification, and activation of compensatory signaling pathways, underscoring the need for molecularly guided treatment sequencing strategies [232,239]. Emerging circulating biomarkers, including microRNAs, may further refine prognostic stratification and treatment selection in endocrine-responsive disease [258].

Looking forward, strategies aimed at overcoming resistance increasingly focus on rational combination approaches and dynamic treatment adaptation. Oral selective estrogen receptor degraders (SERDs), such as camizestrant and elacestrant, offer potential to address ESR1-mediated endocrine resistance, a major driver of disease progression in HR+ breast cancer [239,259]. In parallel, ctDNA analysis is gaining clinical relevance as a minimally invasive tool for detecting resistance mutations, monitoring treatment response, and guiding real-time therapeutic decisions [40].

HER2-targeted therapies exemplify the success of precision oncology in solid tumors. Monoclonal antibodies such as trastuzumab and pertuzumab, together with ADCs, have significantly improved survival outcomes in HER2-positive disease [48,79,111]. Next-generation ADCs, including trastuzumab deruxtecan, extend treatment potential to tumors with low HER2 expression, broadening therapeutic eligibility [26,247]. Despite these advances, treatment-related toxicities—including cardiotoxicity and interstitial lung disease—remain clinically relevant, particularly in heavily pretreated patients. Preclinical HER2-positive breast cancer models continue to improve understanding of therapeutic responses and resistance mechanisms [25,85,260].

The rapid development of antibody–drug conjugates and other next-generation targeted therapies is reshaping the therapeutic landscape of breast cancer. These advances are expected to influence the clinical positioning of established agents such as trastuzumab, abemaciclib, and alpelisib, particularly in terms of treatment sequencing and rational combination strategies. For example, the demonstrated efficacy of HER2-directed ADCs in HER2-low disease may expand the population eligible for HER2-targeted therapy and redefine biomarker thresholds for treatment selection [26,78,79]. Furthermore, improved understanding of resistance mechanisms across HER2, CDK4/6, and PI3K pathways supports a more dynamic, biomarker-driven approach to therapy, including earlier integration of combination regimens or therapeutic switching based on molecular progression [39,261].

The PI3K/AKT/mTOR signaling axis is another critical driver of tumor growth, survival, and therapeutic resistance. The PI3Kα inhibitor alpelisib is clinically approved for PIK3CA-mutated HR+/HER2− advanced breast cancer [212,262], while emerging AKT inhibitors, such as capivasertib, show promising activity in molecularly defined patient subsets. Resistance remains a major challenge, often driven by pathway redundancy, co-occurring genomic alterations, and tumor heterogeneity [247,263]. Interactions between poly(ADP-ribosyl)ation and intracellular signaling events may also contribute to therapeutic resistance, highlighting opportunities for combination strategies [264,265].

Chemotherapy continues to play a central role, particularly in TNBC and high-risk early-stage disease [266]. TNBC is biologically heterogeneous, defined by the absence of ER, PR, and HER2 expression, which historically limited targeted options. Recent advances in immunotherapy, including immune checkpoint inhibitors, have improved outcomes when combined with chemotherapy in both early-stage and metastatic TNBC [22,267]. Emerging strategies now encompass novel cellular therapies, modulation of the tumor microenvironment, and immune checkpoint modulation [268,269,270,271].

PARP inhibitors represent a milestone in biomarker-driven therapy for patients with germline BRCA1/2 mutations, exploiting homologous recombination deficiencies through synthetic lethality [23,272]. Acquired resistance can emerge via restoration of DNA repair, and combination strategies targeting DNA repair or immune pathways may enhance efficacy even in BRCA-proficient tumors [265].

ADCs, including trastuzumab deruxtecan and sacituzumab govitecan, combine antibody specificity with cytotoxic payloads and show significant activity in heavily pretreated patients, including those with HER2-low tumors and metastatic TNBC [26,273]. Advances in understanding tumor antigen expression and molecular heterogeneity have expanded therapeutic indications and improved outcomes [247].

Bone-targeted therapies, including bisphosphonates and denosumab, remain essential in metastatic breast cancer for preventing skeletal-related events and improving quality of life [274].

Emerging therapeutic strategies increasingly focus on overcoming resistance through combination therapies, novel molecular targets, and adaptive treatment approaches. Natural compounds, phytochemicals, and metabolic modulators have demonstrated anticancer effects via apoptosis, cell-cycle arrest, and metabolic disruption [275,276,277]. Advances in nanocarrier-based drug delivery and RNA-targeted therapeutics may further enhance efficacy and overcome chemotherapy resistance [278,279]. Novel small-molecule scaffolds targeting bromodomain-containing proteins involved in transcriptional regulation also show promising preclinical activity [250].

Targeting tumor microenvironment interactions and immune signaling pathways represents a promising approach. Modulation of PD-L1 expression via cytokine signaling may enhance antitumor immunity, while targeting activated pathways involved in epithelial–mesenchymal transition and chemoresistance may improve responses in resistant tumors [280,281]. Cancer stem cells, contributing to recurrence and metastasis, represent additional therapeutic targets [75], and oncolytic virus therapy is being explored to enhance immune responses and treatment outcomes [282].

Advances in computational oncology and artificial intelligence are increasingly being applied to predict treatment responses, identify novel therapeutic targets, and improve clinical trial design in breast cancer research [283]. The integration of genomic profiling, functional biomarkers, and real-time disease monitoring is expected to further refine treatment algorithms and support truly personalized therapeutic strategies across molecular subtypes [24,253,263,284]. In this context, emerging biomarker-driven approaches, including the identification of FOXA1 and RAB25 as predictors of breast cancer cell response to CYP1A1-activated prodrugs, illustrate the growing role of translational molecular insights in guiding precision oncology [285].

Molecular profiling in breast cancer is critically dependent on the quality, timing, and adequacy of biological material obtained for analysis. In the presurgical setting, particularly when neoadjuvant therapy is planned, core needle biopsy specimens must provide sufficient tumor cellularity to enable reliable assessment of key predictive biomarkers, including ER, PR, and HER2, which directly inform the use of HER2-targeted therapies such as trastuzumab, as well as downstream treatment strategies [45,286,287]. In parallel, identification of genomic alterations such as PIK3CA mutations is essential for appropriate selection of PI3K inhibitors, including alpelisib, particularly in hormone receptor–positive disease [70,216]. Pre-analytical variables—including fixation time, tissue handling, and sampling technique—can significantly influence assay performance and should be carefully standardized to minimize the risk of false-negative or discordant biomarker results [288].

In the post-surgical setting, reassessment of biomarker status may be warranted, particularly in cases of residual disease following neoadjuvant therapy, given the potential for tumor heterogeneity and treatment-induced clonal selection [289,290]. This is especially relevant for HER2 expression dynamics, including the emergence of HER2-low phenotypes, as well as for resistance mechanisms affecting CDK4/6 inhibitor efficacy, such as alterations in RB1 or activation of compensatory signaling pathways [36,185]. Biomarker evaluation is routinely performed using IHC for protein expression, ISH for gene amplification, and increasingly through polymerase chain reaction (PCR) and next-generation sequencing (NGS) approaches for the detection of actionable genomic alterations [8,291]. Furthermore, the growing role of liquid biopsy techniques, including ctDNA, offers a minimally invasive strategy for dynamic monitoring of molecular evolution and therapeutic resistance, particularly in the context of CDK4/6 and PI3K pathway–targeted therapies such as abemaciclib and alpelisib [40,41].

Collectively, the integration of high-quality tissue-based and liquid biopsy–derived molecular diagnostics represents a fundamental prerequisite for the effective implementation of precision oncology strategies, enabling optimal patient selection, treatment adaptation, and improved clinical outcomes across HER2-, CDK4/6-, and PI3K-targeted therapeutic paradigms.

**Figure 5:**
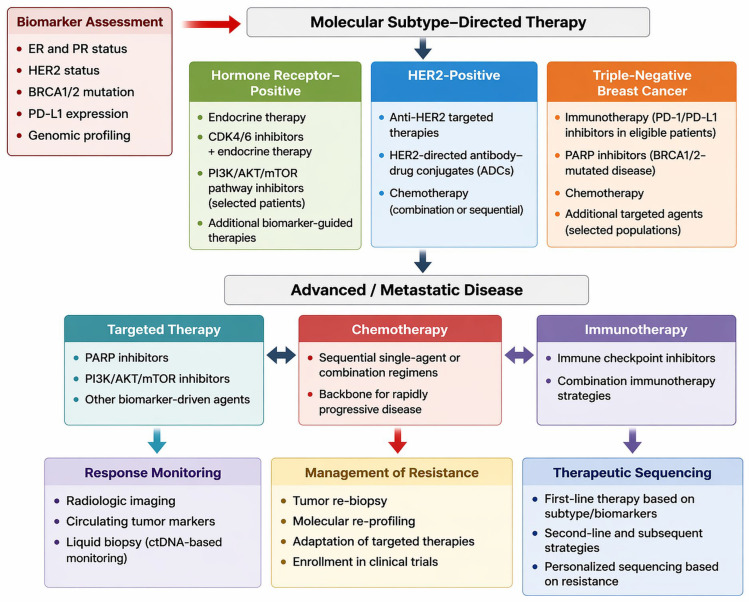
Therapeutic pathways and treatment algorithms in breast cancer integrating biomarkers and treatment sequencing. The presented algorithm represents an interpretative synthesis of current clinical evidence and pivotal trial data, aligned with key principles of NCCN and ESMO guidelines, including biomarker-driven treatment selection and therapy sequencing based on prior exposure and resistance mechanisms. It constitutes an original graphical representation and does not reproduce or replicate any specific published guideline or source. Areas reflecting emerging or investiga-tional strategies are indicated and should be interpreted within the context of evolving clinical evidence. All abbreviations employed are defined in the text in the Abbreviations section.

Table 7 summarizes contemporary management strategies for breast cancer, whereas Figure 5 illustrates therapeutic pathways and treatment algorithms integrating biomarker profiles with treatment sequencing.

## 6. Conclusions

Over the past two decades, breast cancer management has shifted toward precision oncology guided by molecular profiling. Targeted therapies against HER2, CDK4/6, and PI3K pathways have significantly improved outcomes across key subtypes. Trastuzumab has transformed HER2-positive disease, while CDK4/6 inhibitors, such as abemaciclib, have redefined treatment of hormone receptor–positive, HER2-negative cancer. PI3K inhibitors, including alpelisib, further extend personalized treatment options, particularly in PIK3CA-mutant tumors.

Despite these advances, resistance remains a major challenge, underscoring the need for improved biomarkers and combination strategies. Continued integration of genomic profiling, translational research, and real-world evidence will be essential to optimize patient selection and enhance therapeutic durability. Overall, precision medicine approaches are reshaping breast cancer care and continue to drive meaningful improvements in patient outcomes.

## Figures and Tables

**Table 1:** TEAEs and management strategies for trastuzumab according to [45,46,110,129,130,131]. All abbreviations employed are defined in the text in the Abbreviations section.

TEAE	Frequency/ Severity	Timing/ Clinical Features	Recommended Management
Infusion-related reactions (IRR)	Common (~20–40% overall; Grade ≥ 3 rare)	Typically during first infusion; fever, chills, nausea, dyspnea, hypotension, rash	Premedication not routinely required but may be considered; slow/interrupt infusion; symptomatic treatment (antipyretics, antihistamines); discontinue if severe
Cardiotoxicity (↓LVEF, heart failure)	Uncommon but clinically significant (~2–7%; higher with anthracyclines)	Usually asymptomatic LVEF decline; may progress to heart failure; increased risk with prior anthracycline exposure	Baseline and periodic LVEF monitoring; withhold if significant decline; initiate standard heart failure therapy; consider discontinuation if persistent
Hematologic toxicity (including neutropenia, anemia)	Uncommon; more frequent in combination regimens	Often associated with concurrent chemotherapy; neutropenia may increase infection risk	Monitor blood counts; manage per standard oncology guidelines; adjust concomitant chemotherapy if needed
Pulmonary toxicity (including interstitial lung disease, pneumonitis)	Rare but potentially serious	Dyspnea, cough, hypoxia; may occur at any time during treatment	Prompt evaluation; interrupt trastuzumab; corticosteroids if indicated; permanently discontinue in severe cases
Gastrointestinal events (nausea, diarrhea)	Common; usually mild (Grade 1–2)	Occur early during treatment; typically self-limited	Supportive care (antiemetics, antidiarrheals); hydration; no dose modification usually required
General and constitutional symptoms (fatigue, fever)	Common; mostly mild to moderate	Fatigue may persist; fever often infusion-related	Symptomatic management; exclude infection if persistent fever
Hypersensitivity and rare severe reactions (including anaphylaxis, angioedema)	Very rare but potentially life-threatening	Acute onset during or shortly after infusion	Immediate discontinuation; emergency management (epinephrine, corticosteroids, antihistamines); contraindication to rechallenge

**Table 2:** Major pivotal clinical trials of trastuzumab in breast cancer. All abbreviations employed are defined in the text in the Abbreviations section.

Trial	Population	Cancer Setting	Design	Combination	Key Findings	Inclusion/ Eligibility Criteria
HERA [46]	Early HER2+ breast cancer after adjuvant chemotherapy	Adjuvant	Phase III, randomized	Trastuzumab vs. observation	Significant improvement in DFS and OS; established 1-year trastuzumab standard	HER2-positive, completed locoregional therapy and chemotherapy
NSABP B-31/NCCTG N9831 [96]	Early HER2+ breast cancer	Adjuvant	Phase III, randomized	Chemotherapy ± trastuzumab	Marked improvement in DFS and OS with trastuzumab addition	Node-positive or high-risk node-negative HER2+ patients
BCIRG 006 [97]	Early HER2+ breast cancer	Adjuvant	Phase III, randomized	AC→TH vs. TCH vs. AC→T	Comparable efficacy; reduced cardiotoxicity in non-anthracycline arm (TCH)	HER2-positive early-stage breast cancer
NOAH [99]	Locally advanced/inflammatory HER2+ breast cancer	Neoadjuvant	Phase III, randomized	Chemotherapy ± trastuzumab	Increased pCR and event-free survival	HER2+ locally advanced or inflammatory breast cancer
NeoSphere [100]	Early HER2+ breast cancer	Neoadjuvant	Phase II, randomized	Trastuzumab ± pertuzumab + docetaxel	Higher pCR with dual HER2 blockade	HER2-positive, operable, locally advanced, or inflammatory BC
APHINITY [101]	Early HER2+ breast cancer	Adjuvant	Phase III, randomized	Trastuzumab + pertuzumab + chemotherapy vs. trastuzumab + chemotherapy	Improved invasive DFS, especially in node-positive disease	HER2-positive, node-positive or high-risk node-negative
KATHERINE [104]	Residual HER2+ disease after neoadjuvant therapy	Adjuvant (post-neoadjuvant)	Phase III, randomized	T-DM1 vs. trastuzumab	Significantly improved invasive DFS with T-DM1	Residual invasive HER2+ disease after neoadjuvant therapy
CLEOPATRA [110]	Metastatic HER2+ breast cancer	Metastatic (1st-line)	Phase III, randomized	Trastuzumab + pertuzumab + docetaxel	Significant OS and PFS improvement; new standard of care	HER2+ metastatic, no prior anti-HER2 therapy for metastasis
DESTINY-Breast03 [78]	Previously treated HER2+ metastatic breast cancer	Metastatic (2nd-line)	Phase III, randomized	Trastuzumab deruxtecan vs. T-DM1	Markedly improved PFS and OS; established T-DXd as preferred second-line therapy	HER2+, prior trastuzumab and taxane
DESTINY-Breast04 [26]	HER2-low metastatic breast cancer	Metastatic (pretreated)	Phase III, randomized	Trastuzumab deruxtecan vs. physician’s choice chemotherapy	Significant OS and PFS benefit; established HER2-low as a new therapeutic category	HER2-low (IHC 1+ or 2+/ISH−), prior chemotherapy
EMILIA [111]	Previously treated HER2+ metastatic breast cancer	Metastatic (2nd-line)	Phase III, randomized	T-DM1 vs. lapatinib + capecitabine	Improved OS and PFS; better safety profile	HER2+, prior trastuzumab and taxane
M77001 [93]	HER2+ metastatic breast cancer	Metastatic (1st-line)	Phase II/III	Trastuzumab + docetaxel vs. docetaxel	Improved response rate, PFS, and OS	HER2-positive metastatic disease
Slamon trial [45]	HER2+ metastatic breast cancer	Metastatic	Phase III, randomized	Chemotherapy ± trastuzumab	First demonstration of survival benefit with trastuzumab	HER2-overexpressing metastatic breast cancer

**Table 3:** TEAEs and management strategies for abemaciclib according to [57,58,191,192]. All abbreviations employed are defined in the text in the Abbreviations section.

TEAE	Frequency/Severity	Timing/ Clinical Features	Recommended Management
Diarrhea	Very common (~80–85%; Grade ≥ 3~7–10%)	Early onset (often within first week); may be recurrent; dehydration risk if untreated	Initiate antidiarrheal therapy (e.g., loperamide) at first sign; dose interruption/reduction for persistent ≥ Grade 2; ensure hydration and electrolyte monitoring
Neutropenia	Common (~40–50%; Grade ≥ 3 ~20–25%)	Typically occurs during first cycles; less febrile neutropenia than with chemotherapy	Monitor CBC regularly; dose interruption/reduction for ≥Grade 3; G-CSF rarely required
Fatigue	Common (~30–40%; mostly Grade 1–2)	May occur early or persist during treatment	Supportive care; evaluate for contributing factors (e.g., anemia); dose adjustment if severe
Nausea and vomiting	Common (~30–45%; mostly mild–moderate)	Early onset; usually manageable	Antiemetics as needed; dietary modification; maintain hydration
Hepatotoxicity (↑ALT/AST)	Common (~15–20%; Grade ≥ 3 ~5–10%)	Typically within first 2–3 months; often asymptomatic	Monitor liver function tests regularly; dose interruption/reduction for ≥Grade 3; discontinue if severe or persistent
Venous thromboembolism (VTE)	Uncommon but clinically significant (~2–5%)	Deep vein thrombosis or pulmonary embolism; can occur at any time	Initiate anticoagulation; assess risk–benefit for continuation; interrupt or discontinue in severe cases
Interstitial lung disease/pneumonitis	Rare but potentially serious	Dyspnea, cough, hypoxia; variable onset	Interrupt treatment; evaluate promptly; corticosteroids if indicated; discontinue for severe cases
Hematologic toxicity (anemia, thrombocytopenia	Uncommon; usually mild–moderate	Develops during treatment; often asymptomatic	Monitor CBC; supportive care; dose modification if clinically indicated

**Table 4:** Major pivotal clinical trials of abemaciclib in breast cancer. All abbreviations employed are defined in the text in the Abbreviations section.

Trial	Population	Cancer Setting	Design	Combination	Key Findings	Inclusion/ Eligibility Criteria
MONARCH 1 [141]	Heavily pretreated HR+/HER2− metastatic breast cancer	Metastatic (≥2nd-line)	Phase II, single-arm	Abemaciclib monotherapy	Demonstrated clinically meaningful ORR and PFS as monotherapy	HR+/HER2−, prior endocrine therapy and chemotherapy
MONARCH 2 [58]	HR+/HER2− advanced breast cancer progressing on endocrine therapy	Metastatic (1st/2nd-line)	Phase III, randomized	Abemaciclib + fulvestrant vs. placebo + fulvestrant	Significant improvement in PFS and OS	HR+/HER2−, progression on prior endocrine therapy
MONARCH 3 [57]	HR+/HER2− advanced breast cancer (endocrine therapy-naïve)	Metastatic (1st-line)	Phase III, randomized	Abemaciclib + aromatase inhibitor vs. placebo + AI	Significant improvement in PFS	Postmenopausal women, HR+/HER2−, no prior systemic therapy for advanced disease
MONARCH plus [193]	HR+/HER2− advanced breast cancer (Asian population)	Metastatic	Phase III, randomized	Abemaciclib + endocrine therapy vs. placebo + endocrine therapy	Confirmed PFS benefit in Asian population	HR+/HER2− advanced breast cancer
monarchE [194]	High-risk early HR+/HER2− breast cancer	Adjuvant	Phase III, randomized	Abemaciclib + endocrine therapy vs. endocrine therapy alone	Improved invasive DFS; established adjuvant role in high-risk patients	HR+/HER2−, node-positive, high-risk features (e.g., Ki-67 ≥ 20%)
nextMONARCH [195]	HR+/HER2− metastatic breast cancer	Metastatic (pretreated)	Phase II, randomized	Abemaciclib ± tamoxifen	Improved PFS with combination; dose/schedule optimization	HR+/HER2−, prior chemotherapy

**Table 5:** TEAEs and management strategies for alpelisib according to [70,243,244]. All abbreviations employed are defined in the text in the Abbreviations section.

TEAE	Frequency/Severity	Timing/ Clinical Features	Recommended Management
Hyperglycemia	Very common (~60–65%; Grade ≥ 3~30–40%)	Early onset (often within first 2 weeks); may be severe; risk higher in prediabetes/diabetes	Baseline and frequent glucose monitoring; initiate metformin as first-line; add insulin or other agents if needed; dose interruption/reduction for ≥Grade 3
Rash (maculopapular)	Common (~35–40%; Grade ≥ 3~10%)	Typically within first 2–3 weeks; may be pruritic	Prophylactic antihistamines may reduce incidence; topical/systemic corticosteroids; dose interruption/reduction for ≥Grade 2–3
Diarrhea	Common (~55–60%; Grade ≥ 3~5–10%)	Early onset; may lead to dehydration if untreated	Antidiarrheal agents (e.g., loperamide); hydration; dose modification for persistent or severe cases
Stomatitis/mucositis	Common (~25–30%; mostly Grade 1–2)	Occurs early during treatment	Good oral hygiene; topical corticosteroids or analgesics; dose adjustment if severe
Fatigue	Common (~30–40%; mostly mild–moderate)	May persist throughout therapy	Supportive care; assess for contributing factors; dose adjustment if severe
Hepatotoxicity (↑ALT/AST)	Uncommon (~10–15%; Grade ≥ 3~5%)	Usually within first months; often asymptomatic	Monitor liver function tests; dose interruption/reduction for ≥Grade 3; discontinue if severe
Non-infectious pneumonitis/ILD	Rare but potentially serious	Dyspnea, cough, hypoxia; variable onset	Interrupt treatment; evaluate promptly; corticosteroids if indicated; discontinue if confirmed and severe
Hypersensitivity and severe cutaneous reactions (e.g., SJS/TEN grouped)	Very rare but life-threatening	Severe rash, mucosal involvement, systemic symptoms	Immediate discontinuation; urgent specialist care; contraindication to rechallenge

**Table 6:** Major pivotal clinical trials of alpelisib in breast cancer, including taselisib and buparlisib to illustrate class effects and PI3K pathway targeting. All abbreviations employed are defined in the text in the Abbreviations section.

Trial	Population	Cancer Setting	Design	Combination	Key Findings	Inclusion/ Eligibility Criteria
SOLAR-1 [70]	HR+/HER2− advanced breast cancer with PIK3CA mutation	Metastatic (1st/2nd-line)	Phase III, randomized	Alpelisib + fulvestrant vs. placebo + fulvestrant	Significant improvement in PFS in PIK3CA-mutant cohort; established biomarker-driven therapy	HR+/HER2−, PIK3CA-mutated, progression on or after endocrine therapy
BYLieve [166]	HR+/HER2− advanced breast cancer with PIK3CA mutation after CDK4/6 inhibitor	Metastatic (post-CDK4/6i)	Phase II, non-randomized	Alpelisib + endocrine therapy (fulvestrant or AI)	Clinically meaningful activity post-CDK4/6 inhibitor; supports real-world sequencing	HR+/HER2−, PIK3CA-mutated, prior CDK4/6 inhibitor
BELLE-2 [245]	HR+/HER2− advanced breast cancer	Metastatic	Phase III, randomized	Buparlisib + fulvestrant vs. placebo + fulvestrant	Improved PFS in PIK3CA-altered tumors; limited by toxicity	HR+/HER2−, progression on aromatase inhibitor
BELLE-3 [175]	HR+/HER2− advanced breast cancer after mTOR inhibitor	Metastatic	Phase III, randomized	Buparlisib + fulvestrant vs. placebo + fulvestrant	Improved PFS; highlights PI3K pathway relevance after mTOR resistance	HR+/HER2−, prior mTOR inhibitor
SANDPIPER [173]	HR+/HER2− advanced breast cancer (PIK3CA-mutant subgroup)	Metastatic	Phase III, randomized	Taselisib + fulvestrant vs. placebo + fulvestrant	Modest PFS benefit; higher toxicity; supports class effect of PI3K inhibition	HR+/HER2−, PIK3CA-mutated, prior endocrine therapy

**Table 7:** Contemporary management of breast cancer: clinically focused overview of current treatment strategies, including indications, representative regimens, biomarkers, toxicity considerations, and supporting evidence. All abbreviations employed are defined in the text in the Abbreviations section.

Modality	Indication/ When Used	Example Regimens/Agents	Key Evidence	Biomarkers/ Toxicity
Surgery	Early-stage (I–III); selected oligometastatic disease	Breast-conserving surgery + SLNB; mastectomy ± reconstruction	NSABP B-06: survival equivalence of BCS + RT vs. mastectomy [251]	ER/PR/HER2 guide adjuvant therapy; lymphedema, surgical complications
Radiotherapy	Adjuvant after BCS; post-mastectomy (node+ or high risk); palliation	Whole-breast RT ± boost; regional nodal irradiation	EBCTCG meta-analyses: ↓ recurrence and mortality [252]	No predictive biomarkers; dermatitis, fibrosis, rare cardiotoxicity
Endocrine therapy (ET)	HR+ early and metastatic disease	Tamoxifen; aromatase inhibitors (letrozole, anastrozole); fulvestrant	ATAC, BIG 1-98: improved DFS/OS [254,255]	ER/PR+; menopausal symptoms, osteoporosis, thromboembolism
CDK4/6 inhibitors + ET	First-line HR+/HER2− advanced/metastatic	Abemaciclib, palbociclib, ribociclib + AI or fulvestrant	MONARCH, PALOMA, MONALEESA: improved PFS/OS [58,151,292]	ER+, intact Rb; neutropenia (palbociclib), diarrhea (abemaciclib), QT prolongation (ribociclib)
HER2-targeted therapy	HER2+ early and metastatic disease	Trastuzumab ± pertuzumab; T-DM1; trastuzumab deruxtecan; neratinib	CLEOPATRA, EMILIA, DESTINY-Breast [48,79,111]	HER2 amplification/overexpression; cardiotoxicity, ILD (T-DXd), diarrhea
Chemotherapy	Neoadjuvant (TNBC, HER2+); adjuvant (high risk); metastatic	Anthracycline + taxane (AC→T); carboplatin (TNBC)	Standard backbone; improves pCR and survival [293]	TNBC subtype; myelosuppression, neuropathy, cardiotoxicity
Immunotherapy	PD-L1+ metastatic TNBC; high-risk early TNBC (neoadjuvant)	Pembrolizumab + chemotherapy; atezolizumab + nab-paclitaxel	KEYNOTE-355, KEYNOTE-522 [267,294]	PD-L1 expression; immune-related AEs (thyroiditis, pneumonitis)
PARP inhibitors	gBRCA1/2-mutated HER2− metastatic or high-risk early	Olaparib, talazoparib	OlympiAD, EMBRACA [23,272]	Germline BRCA mutations; anemia, fatigue, nausea
PI3K/AKT pathway inhibitors	HR+/HER2− metastatic with pathway alterations after ET	Alpelisib + fulvestrant; capivasertib + fulvestrant	SOLAR-1, CAPItello-291 [70,262]	PIK3CA, AKT1, PTEN; hyperglycemia, rash, diarrhea
Antibody–drug conjugates (ADCs)	HER2+ and HER2-low metastatic; pretreated disease	Trastuzumab deruxtecan; sacituzumab govitecan	DESTINY-Breast04; ASCENT [273,295]	HER2 (incl. low); ILD, neutropenia
Bone-targeted therapy	Bone metastases	Zoledronic acid; denosumab	Reduce skeletal-related events [274]	No specific biomarkers; osteonecrosis of jaw, hypocalcemia
Emerging/precision strategies	Resistance-driven sequencing; biomarker-adapted therapy	Oral SERDs (e.g., camizestrant); novel combinations	Ongoing biomarker-driven trials [296]	ESR1 mutations, ctDNA; class-specific toxicities

## Data Availability

No new data were created or analyzed in this study. Data sharing is not applicable to this article.

## Abbreviations

The following abbreviations are used in this manuscript:

AC	doxorubicin (Adriamycin) plus cyclophosphamide
ADC	antibody–drug conjugate
ADCC	antibody-dependent cellular cytotoxicity
AI	aromatase inhibitor
AKT	protein kinase B (AKT serine/threonine kinase)
ALT	alanine aminotransferase
APHINITY	adjuvant pertuzumab and trastuzumab in initial therapy
AST	aspartate aminotransferase
B-31/9831	National Surgical Adjuvant Breast and Bowel Project (NSABP) B-31/North Central Cancer Treatment Group (NCCTG) N9831 trials
BC	breast cancer
BCIRG	Breast Cancer International Research Group
BCS	breast-conserving surgery
BRCA	breast cancer gene (BRCA1/2)
CBC	complete blood count
CDK2	cyclin-dependent kinase 2
CDK4/6	cyclin-dependent kinases 4 and 6
Cip/Kip	CDK-interacting protein/kinase inhibitory protein family
CLEOPATRA	clinical evaluation of pertuzumab and trastuzumab
ctDNA	circulating tumor DNA
DESTINY-Breast03/04	trastuzumab deruxtecan clinical trials in breast cancer
DFS	disease-free survival
E2F	E2F transcription factor (E2 factor/promoter of adenovirus genes)
EFS	event-free survival
EGFR	epidermal growth factor receptor
EMILIA	T-DM1 vs. lapatinib plus capecitabine trial
ER	estrogen receptor
ET	endocrine therapy
FcR	Fc receptor
FGFR1	fibroblast growth factor receptor 1
G1	gap 1 phase
G2	gap 2 phase
HERA	Herceptin Adjuvant Trial
HER2(+/−)	human epidermal growth factor receptor 2 (-positive/negative)
HER2-low	HER2 low expression (IHC 1+ or IHC 2+/ISH−)
HR+	hormone receptor positive
Hippo pathway	Hippo signaling pathway
IHC	immunohistochemistry
ILD	interstitial lung disease
IO	immuno-oncology
IRR	infusion-related reactions
ISH	in situ hybridization
KATHERINE	trastuzumab emtansine vs. trastuzumab in residual disease
Ki-67	proliferation marker
LVEF	left ventricular ejection fraction
MAPK	mitogen-activated protein kinase
M	mitosis phase
monarchE	abemaciclib in high-risk early breast cancer
mTOR	mechanistic target of rapamycin
NeoSphere	neoadjuvant study of pertuzumab and trastuzumab
NOAH	neoadjuvant Herceptin trial
NR	not reported
ORR	objective response rate
OS	overall survival
p16	cyclin-dependent kinase inhibitor 2A (CDKN2A)
p21	cyclin-dependent kinase inhibitor 1A (CDKN1A)
p27	cyclin-dependent kinase inhibitor 1B (CDKN1B)
p85	regulatory subunit of PI3K
p110	catalytic subunit of PI3K
p110α	catalytic subunit alpha isoform of PI3K
PARP	poly(ADP-ribose) polymerase
pCR	pathologic complete response
PD-L1	programmed death-ligand 1
PFS	progression-free survival
PI3K(α/CA)	phosphatidylinositol-3-kinase (alpha isoform/catalytic subunit alpha)
PIP2	phosphatidylinositol 4,5-bisphosphate
PIP3	phosphatidylinositol 3,4,5-trisphosphate
PR	progesterone receptor
PTEN	phosphatase and tensin homolog
RAS	rat sarcoma small GTPase
Rb	retinoblastoma protein
pRb	retinoblastoma protein
RT	radiotherapy
S	synthesis phase
SERD(s)	selective estrogen receptor degrader(s)
SRC	SRC proto-oncogene tyrosine kinase
SJS	Stevens–Johnson syndrome
SLNB	sentinel lymph node biopsy
T	docetaxel (taxane chemotherapy drug)
TCH	docetaxel, carboplatin, and trastuzumab
T-DM1	trastuzumab emtansine
T-DXd	trastuzumab deruxtecan
TEAE	treatment-emergent adverse event
TEN	toxic epidermal necrolysis
TH	docetaxel plus trastuzumab
TNBC	triple-negative breast cancer
VTE	venous thromboembolism

## References

[1] Ben-Dror J., Shalamov M., Sonnenblick A. (2022). The history of early breast cancer treatment. Genes.

[2] Sung H., Ferlay J., Siegel R.L., Laversanne M., Soerjomataram I., Jemal A., Bray F. (2021). Global Cancer Statistics 2020: GLOBOCAN estimates of incidence and mortality worldwide for 36 cancers in 185 countries. CA Cancer J. Clin..

[3] Siegel R.L., Miller K.D., Wagle N.S., Jemal A. (2023). Cancer statistics, 2023. CA Cancer J. Clin..

[4] Ye F., Dewanjee S., Li Y., Jha N.K., Chen Z.S., Kumar A., Vishakha, Behl T., Jha S.K., Tang H. (2023). Advancements in clinical aspects of targeted therapy and immunotherapy in breast cancer. Mol. Cancer.

[5] Hong R., Xu B. (2022). Breast cancer: An up-to-date review and future perspectives. Cancer Commun..

[6] Perou C.M., Sørlie T., Eisen M.B., van de Rijn M., Jeffrey S.S., Rees C.A., Pollack J.R., Ross D.T., Johnsen H., Akslen L.A. (2000). Molecular portraits of human breast tumours. Nature.

[7] Sørlie T., Perou C.M., Tibshirani R., Aas T., Geisler S., Johnsen H., Hastie T., Eisen M.B., van de Rijn M., Jeffrey S.S. (2001). Gene expression patterns of breast carcinomas distinguish tumor subclasses with clinical implications. Proc. Natl. Acad. Sci. USA.

[8] Cancer Genome Atlas Network (2012). Comprehensive molecular portraits of human breast tumours. Nature.

[9] Liang Y., Zhang H., Song X., Yang Q. (2020). Metastatic heterogeneity of breast cancer: Molecular mechanism and potential therapeutic targets. Semin. Cancer Biol..

[10] Xiong X., Zheng L.W., Ding Y., Chen Y.F., Cai Y.W., Wang L.P., Huang L., Liu C.C., Shao Z.M., Yu K.D. (2025). Breast cancer: Pathogenesis and treatments. Signal Transduct. Target. Ther..

[11] Prat A., Pineda E., Adamo B., Galván P., Fernández A., Gaba L., Díez M., Viladot M., Arance A., Muñoz M. (2015). Clinical implications of the intrinsic molecular subtypes of breast cancer. Breast.

[12] Baselga J., Swain S.M. (2009). Novel anticancer targets: Revisiting ERBB2 and discovering ERBB3. Nat. Rev. Cancer.

[13] Arteaga C.L., Sliwkowski M.X., Osborne C.K., Perez E.A., Puglisi F., Gianni L. (2012). Treatment of HER2-positive breast cancer: Current status and future perspectives. Nat. Rev. Clin. Oncol..

[14] Turner N.C., Reis-Filho J.S. (2012). Genetic heterogeneity and cancer drug resistance. Lancet Oncol..

[15] Jacobs A.T., Martinez Castaneda-Cruz D., Rose M.M., Connelly L. (2022). Targeted therapy for breast cancer: An overview of drug classes and outcomes. Biochem. Pharmacol..

[16] Mohamed A., Krajewski K., Cakar B., Ma C.X. (2013). Targeted therapy for breast cancer. Am. J. Pathol..

[17] Gu G., Dustin D., Fuqua S.A. (2016). Targeted therapy for breast cancer and molecular mechanisms of resistance to treatment. Curr. Opin. Pharmacol..

[18] Goldhirsch A., Winer E.P., Coates A.S., Gelber R.D., Piccart-Gebhart M., Thürlimann B., Senn H.-J., Panel Members (2013). Personalizing the treatment of women with early breast cancer: Highlights of the St Gallen International Expert Consensus on the Primary Therapy of Early Breast Cancer 2013. Ann. Oncol..

[19] Prat A., Cheang M.C.U., Martín M., Parker J.S., Carrasco E., Caballero R., Tyldesley S., Gelmon K., Bernard P.S., Nielsen T.O. (2013). Prognostic significance of progesterone receptor-positive tumor cells within immunohistochemically defined luminal A breast cancer. J. Clin. Oncol..

[20] Won K.A., Spruck C. (2020). Triple-negative breast cancer therapy: Current and future perspectives. Int. J. Oncol..

[21] Li Y., Zhang H., Merkher Y., Chen L., Liu N., Leonov S., Chen Y. (2022). Recent advances in therapeutic strategies for triple-negative breast cancer. J. Hematol. Oncol..

[22] Schmid P., Adams S., Rugo H.S., Schneeweiss A., Barrios C.H., Iwata H., Diéras V., Hegg R., Im S.A., Shaw Wright G. (2018). Atezolizumab and nab-paclitaxel in advanced triple-negative breast cancer. N. Engl. J. Med..

[23] Robson M., Im S.-A., Senkus E., Xu B., Domchek S.M., Masuda N., Delaloge S., Li W., Tung N., Armstrong A. (2017). Olaparib for metastatic breast cancer in patients with a germline BRCA mutation. N. Engl. J. Med..

[24] Bianchini G., Balko J.M., Mayer I.A., Sanders M.E., Gianni L. (2016). Triple-negative breast cancer: Challenges and opportunities of a heterogeneous disease. Nat. Rev. Clin. Oncol..

[25] Kang S., Kim S.B. (2024). HER2-Low Breast Cancer: Now and in the Future. Cancer Res. Treat..

[26] Modi S., Jacot W., Yamashita T., Sohn J., Vidal M., Tokunaga E., Tsurutani J., Ueno N.T., Prat A., Chae Y.S. (2022). Trastuzumab Deruxtecan in Previously Treated HER2-Low Advanced Breast Cancer. N. Engl. J. Med..

[27] Tarantino P., Hamilton E., Tolaney S.M., Cortes J., Morganti S., Ferraro E., Marra A., Viale G., Trapani D., Cardoso F. (2020). HER2-Low Breast Cancer: Pathological and Clinical Landscape. J. Clin. Oncol..

[28] Yarden Y., Sliwkowski M.X. (2001). Untangling the ErbB signalling network. Nat. Rev. Mol. Cell Biol..

[29] Sherr C.J., Roberts J.M. (1999). CDK inhibitors: Positive and negative regulators of G1-phase progression. Genes Dev..

[30] Fruman D.A., Chiu H., Hopkins B.D., Bagrodia S., Cantley L.C., Abraham R.T. (2017). The PI3K pathway in human disease. Cell.

[31] Cheng X. (2024). A comprehensive review of HER2 in cancer biology and therapeutics. Genes.

[32] Lawler M., Keeling P., Kholmanskikh O., Minnaard W., Moehlig-Zuttermeister H., Normanno N., Philip R., Popp C., Salgado R., Santiago-Walker A.E. (2024). Empowering effective biomarker-driven precision oncology: A call to action. Eur. J. Cancer.

[33] Quinn R., Patel R., Sison C., Singh A., Zhu X.-H. (2021). Impact of precision medicine on clinical outcomes: A single-institution retrospective study. Front. Oncol..

[34] Tsimberidou A.M., Eggermont A.M.M., Schilsky R.L. (2014). Precision cancer medicine: The future is now, only better. Am. Soc. Clin. Oncol. Educ. Book.

[35] Sarhangi N., Hajjari S., Heydari S.F., Ganjizadeh M., Rouhollah F., Hasanzad M. (2022). Breast cancer in the era of precision medicine. Mol. Biol. Rep..

[36] O’Leary B., Finn R.S., Turner N.C. (2016). Treating cancer with selective CDK4/6 inhibitors. Nat. Rev. Clin. Oncol..

[37] Formisano L., Lu Y., Servetto A., Hanker A.B., Jansen V.M., Bauer J.A., Sudhan D.R., Guerrero-Zotano A.L., Croessmann S., Guo Y. (2019). Aberrant FGFR signaling mediates resistance to CDK4/6 inhibitors in ER+ breast cancer. Nat. Commun..

[38] Wander S.A., Cohen O., Gong X., Johnson G.N., Buendia-Buendia J.E., Lloyd M.R., Kim D., Luo F., Mao P., Helvie K. (2020). The genomic landscape of intrinsic and acquired resistance to cyclin-dependent kinase 4/6 inhibitors in patients with hormone receptor-positive metastatic breast cancer. Cancer Discov..

[39] Condorelli R., Spring L., O’Shaughnessy J., Lacroix L., Bailleux C., Scott V., Dubois J., Nagy R.J., Lanman R.B., Iafrate A.J. (2018). Polyclonal RB1 mutations and acquired resistance to CDK4/6 inhibitors in patients with metastatic breast cancer. Ann. Oncol..

[40] Dawson S.-J., Tsui D.W.Y., Murtaza M., Biggs H., Rueda O.M., Chin S.-F., Dunning M.J., Gale D., Forshew T., Mahler-Araujo B. (2013). Analysis of circulating tumor DNA to monitor metastatic breast cancer. N. Engl. J. Med..

[41] Turner N.C., Kingston B., Kilburn L.S., Kernaghan S., Wardley A.M., Macpherson I.R., Baird R.D., Roylance R., Stephens P., Oikonomidou O. (2020). Circulating tumour DNA analysis to direct therapy in advanced breast cancer (plasmaMATCH): A multicentre, multicohort, phase 2a, platform trial. Lancet Oncol..

[42] Seo J., Koh J., Lee D.-W., Kim J., Ryu H.S., Lee K.-H., Kim T.-Y., Im S.-A. (2023). HER2 amplification level by in situ hybridization predicts survival outcome in advanced HER2-positive breast cancer treated with pertuzumab, trastuzumab, and docetaxel regardless of HER2 IHC results. Breast Cancer Res..

[43] Wolff A.C., Somerfield M.R., Dowsett M., Hammond M.E.H., Hayes D.F., McShane L.M., Saphner T.J., Spears P.A., Allison K.H. (2023). Human epidermal growth factor receptor 2 testing in breast cancer: ASCO-College of American Pathologists guideline update. J. Clin. Oncol..

[44] Kunte S., Abraham J., Montero A.J. (2020). Novel HER2-targeted therapies for HER2-positive metastatic breast cancer. Cancer.

[45] Slamon D.J., Leyland-Jones B., Shak S., Fuchs H., Paton V., Bajamonde A., Fleming T., Eiermann W., Wolter J., Pegram M. (2001). Use of chemotherapy plus a monoclonal antibody against HER2 for metastatic breast cancer that overexpresses HER2. N. Engl. J. Med..

[46] Piccart-Gebhart M.J., Procter M., Leyland-Jones B., Goldhirsch A., Untch M., Smith I., Gianni L., Baselga J., Bell R., Jackisch C. (2005). Trastuzumab after adjuvant chemotherapy in HER2-positive breast cancer. N. Engl. J. Med..

[47] Aitelhaj M., Lkhoyaali S., Rais G., Boutayeb S., Errihani H. (2016). First line chemotherapy plus trastuzumab in metastatic breast cancer HER2 positive—Observational institutional study. Pan Afr. Med. J..

[48] Swain S.M., Miles D., Kim S.B., Im Y.H., Im S.A., Semiglazov V., Ciruelos E., Schneeweiss A., Loi S., Monturus E. (2020). Pertuzumab, trastuzumab, and docetaxel for HER2-positive metastatic breast cancer (CLEOPATRA): End-of-study results from a double-blind, randomised, placebo-controlled, phase 3 study. Lancet Oncol..

[49] Valabrega G., Montemurro F., Aglietta M. (2007). Trastuzumab: Mechanism of action, resistance and future perspectives in HER2-overexpressing breast cancer. Ann. Oncol..

[50] Vivekanandhan S., Knutson K.L. (2022). Resistance to Trastuzumab. Cancers.

[51] Wang L., Wang Y., Li Y., Zhou L., Du J., Wang J., Liu S., Cao Y., Li Y., Yang W. (2024). Resistance mechanisms and prospects of trastuzumab. Front. Oncol..

[52] Clynes R.A., Towers T.L., Presta L.G., Ravetch J.V. (2000). Inhibitory Fc receptors modulate in vivo cytotoxicity against tumor targets. Nat. Med..

[53] Shang Q., Lin Z., Plichta J., Thomas S., Ouyang M., Luo S., Wang X. (2026). Comprehensive analysis and prediction of HER2-targeted therapy insensitivity among HER2-positive breast cancer patients undergoing neoadjuvant treatment. Cancers.

[54] Finn R.S., Martin M., Rugo H.S., Jones S., Im S.A., Gelmon K., Harbeck N., Lipatov O.N., Walshe J.M., Moulder S. (2016). Palbociclib and Letrozole in Advanced Breast Cancer. N. Engl. J. Med..

[55] Matthews H.K., Bertoli C., de Bruin R.A.M. (2022). Cell cycle control in cancer. Nat. Rev. Mol. Cell Biol..

[56] Gao T., Sun Y., Leng P., Liu D., Guo Q., Li J. (2025). CDK4/6 inhibitors in breast cancer therapy: Mechanisms of drug resistance and strategies for treatment. Front. Pharmacol..

[57] Goetz M.P., Toi M., Campone M., Sohn J., Paluch-Shimon S., Huober J., Park I.H., Trédan O., Chen S.-C., Manso L. (2017). MONARCH 3: Abemaciclib as initial therapy for advanced breast cancer. J. Clin. Oncol..

[58] Sledge G.W., Toi M., Neven P., Sohn J., Inoue K., Pivot X., Burdaeva O., Okera M., Masuda N., Kaufman P.A. (2017). MONARCH 2: Abemaciclib in combination with fulvestrant in women with HR+/HER2− advanced breast cancer who had progressed while receiving endocrine therapy. J. Clin. Oncol..

[59] Johnston S., Martin M., O’Shaughnessy J., Hegg R., Tolaney S.M., Guarneri V., Del Mastro L., Campone M., Sohn J., Boyle F. (2026). Overall survival with abemaciclib in early breast cancer. Ann. Oncol..

[60] Lloyd M.R., Jhaveri K., Kalinsky K., Bardia A., Wander S.A. (2024). Precision therapeutics and emerging strategies for HR-positive metastatic breast cancer. Nat. Rev. Clin. Oncol..

[61] Pavlovic D., Niciforovic D., Papic D., Milojevic K., Markovic M. (2023). CDK4/6 inhibitors: Basics, pros, and major cons in breast cancer treatment with specific regard to cardiotoxicity—A narrative review. Ther. Adv. Med. Oncol..

[62] Shanabag A., Armand J., Son E., Yang H.W. (2025). Targeting CDK4/6 in breast cancer. Exp. Mol. Med..

[63] Glaviano A., Wander S.A., Baird R.D., Yap K.C.-H., Lam H.Y., Toi M., Carbone D., Geoerger B., Serra V., Jones R.H. (2024). Mechanisms of sensitivity and resistance to CDK4/CDK6 inhibitors in hormone receptor-positive breast cancer treatment. Drug Resist. Updat..

[64] Wang Y., Rozen V., Zhao Y., Wang Z. (2024). Oncogenic activation of PI3KCA in cancers: Emerging targeted therapies in precision oncology. Genes Dis..

[65] He Y., Sun M.M., Zhang G.G., Yang J., Chen K.S., Xu W.W., Li B. (2021). Targeting PI3K/Akt signal transduction for cancer therapy. Signal Transduct. Target Ther..

[66] Hossain M.T., Hossain M.A. (2025). Targeting PI3K in cancer treatment: A comprehensive review with insights from clinical outcomes. Eur. J. Pharmacol..

[67] Mukohara T. (2015). PI3K mutations in breast cancer: Prognostic and therapeutic implications. Breast Cancer.

[68] LoRusso P.M. (2016). Inhibition of the PI3K/AKT/mTOR Pathway in Solid Tumors. J. Clin. Oncol..

[69] Juric D., Rodon J., Tabernero J., Janku F., Burris H.A., Schellens J.H.M., Middleton M.R., Berlin J., Schuler M., Gil-Martin M. (2018). Phosphatidylinositol 3-Kinase α-Selective Inhibition With Alpelisib (BYL719) in PIK3CA-Altered Solid Tumors: Results From the First-in-Human Study. J. Clin. Oncol..

[70] André F., Ciruelos E., Rubovszky G., Campone M., Loibl S., Rugo H.S., Iwata H., Conte P., Mayer I.A., Kaufman B. (2019). Alpelisib for PIK3CA-Mutated, Hormone Receptor-Positive Advanced Breast Cancer. N. Engl. J. Med..

[71] Mosele M.F., Westphalen C.B., Stenzinger A., Barlesi F., Bayle A., Bièche I., Bonastre J., Castro E., Dienstmann R., Krämer A. (2024). Recommendations for the use of next-generation sequencing (NGS) for patients with advanced cancer in 2024: A report from the ESMO Precision Medicine Working Group. Ann. Oncol..

[72] Lev S. (2020). Targeted therapy and drug resistance in triple-negative breast cancer: The EGFR axis. Biochem. Soc. Trans..

[73] Harris M.A., Savas P., Virassamy B., O’Malley M.M.R., Kay J., Mueller S.N., Mackay L.K., Salgado R., Loi S. (2024). Towards targeting the breast cancer immune microenvironment. Nat. Rev. Cancer.

[74] Vijayakumar S., Dhakshanamoorthy R., Baskaran A., Krishnan B.S., Maddaly R. (2024). Drug resistance in human cancers—Mechanisms and implications. Life Sci..

[75] Walcher L., Kistenmacher A.K., Suo H., Kitte R., Dluczek S., Strauß A., Blaudszun A.R., Yevsa T., Fricke S., Kossatz-Boehlert U. (2020). Cancer Stem Cells—Origins and Biomarkers: Perspectives for Targeted Personalized Therapies. Front. Immunol..

[76] Loi S., Giobbie-Hurder A., Gombos A., Bachelot T., Hui R., Curigliano G., Campone M., Biganzoli L., Bonnefoi H., Jerusalem G. (2019). Pembrolizumab plus trastuzumab in trastuzumab-resistant, advanced, HER2-positive breast cancer (PANACEA): A single-arm, multicentre, phase 1b–2 trial. Lancet Oncol..

[77] Emens L.A., Esteva F.J., Beresford M., Saura C., De Laurentiis M., Kim S.-B., Im S.-A., Wang Y., Salgado R., Mani A. (2020). Trastuzumab emtansine plus atezolizumab versus trastuzumab emtansine plus placebo in previously treated, HER2-positive advanced breast cancer (KATE2): A phase 2, multicentre, randomised, double-blind trial. Lancet Oncol..

[78] Cortés J., Kim S.-B., Chung W.-P., Im S.-A., Park Y.H., Hegg R., Kim M.H., Tseng L.-M., Petry V., Chung C.-F. (2022). Trastuzumab Deruxtecan versus Trastuzumab Emtansine for Breast Cancer. N. Engl. J. Med..

[79] Modi S., Saura C., Yamashita T., Park Y.H., Kim S.-B., Tamura K., André F., Iwata H., Ito Y., Tsurutani J. (2020). Trastuzumab deruxtecan in previously treated HER2-positive breast cancer. N. Engl. J. Med..

[80] Baselga J., Albanell J., Molina M.A., Arribas J. (2001). Mechanism of action of trastuzumab and scientific update. Semin. Oncol..

[81] Hudis C.A. (2007). Trastuzumab—Mechanism of action and use in clinical practice. N. Engl. J. Med..

[82] Maadi H., Soheilifar M.H., Choi W.-S., Moshtaghian A., Wang Z. (2021). Trastuzumab mechanism of action: 20 years of research to unravel a dilemma. Cancers.

[83] Boz A.B., Er I., Picher E.A., Smarakan S. (2026). AXL-Driven Stemness and Hedgehog Signaling in HER2-Positive Breast Cancer with Acquired Trastuzumab Resistance: Synergistic Potential of AXL and HER2 Co-Targeting. Life.

[84] Yamamoto Y. (2026). Overcoming Trastuzumab-Pertuzumab Resistance and Optimizing Sequential Anti-HER2 Therapy in HER2-Positive Metastatic Breast Cancer. Cancers.

[85] Lu Y., Lee B.P., Eli A.V., Lynch S.E., Faisal A.R.M., Moye J., Sorace A.G. (2026). Characterization of HER2-Positive Murine Breast Cancer Models for Investigating HER2-Targeted Therapy and Immunotherapy. Cancers.

[86] Dursun B., Yaslıkaya Ş., Sekmek S., Önder T., Yıldırım H.Ç., Acar Ö., Sever N., Gökçek S., Ön S., Aslan F. (2026). Clinical Significance of Achieving No Evidence of Disease in HER2-Positive Metastatic Breast Cancer: A Multicenter Study by Turkish Oncology Group (TOG). Cancers.

[87] Nahta R., Yu D., Hung M.-C., Hortobagyi G.N., Esteva F.J. (2006). Mechanisms of disease: Understanding resistance to HER2-targeted therapy in human breast cancer. Nat. Clin. Pract. Oncol..

[88] Rexer B.N., Arteaga C.L. (2012). Intrinsic and acquired resistance to HER2-targeted therapies in HER2 gene-amplified breast cancer: Mechanisms and clinical implications. Crit. Rev. Oncog..

[89] Bayramgil A., Yücel M.H., Turkoglu E., Guren A.K., Kemik F., Ulufer B., Çakan Demirel B., Yildiz A., Sacli O., Ercin E. (2026). Real-World Outcomes of Neoadjuvant Dual Blockade in HER2-Positive Breast Cancer: The Role of Tumor Biology and pCR. J. Clin. Med..

[90] Trastuzumab Overview. Mechanism of Action of Trastuzumab. https://www.creativebiolabs.net/trastuzumab-overview.htm.

[91] Burstein H.J. (2005). The distinctive nature of HER2-positive breast cancers. N. Engl. J. Med..

[92] Denkert C., Liedtke C., Tutt A., von Minckwitz G. (2017). Molecular Alterations in Triple-Negative Breast Cancer—The Road to New Treatment Strategies. Lancet.

[93] Marty M., Cognetti F., Maraninchi D., Snyder R., Mauriac L., Tubiana-Hulin M., Chan S., Grimes D., Antón A., Lluch A. (2005). Randomized phase II trial of the efficacy and safety of trastuzumab combined with docetaxel in patients with human epidermal growth factor receptor 2-positive metastatic breast cancer administered as first-line treatment: The M77001 study group. J. Clin. Oncol..

[94] Irelli A., Sidoni T., Pavese F., Rotondaro S., Luzi C., Zelli V., Centonze S., Patruno L.V., Zazzeroni F., Tessitore A. (2026). Anthracycline-Free Neoadjuvant Pertuzumab-Trastuzumab-Taxane in Patients with HER2-Positive Early Breast Cancer: Hormone Receptor Status as a Key Determinant of Pathological Complete Response. Biomedicines.

[95] Pivot X., Suter T., Nabholtz J.M., Pierga J.Y., Espie M., Lortholary A., Khayat D., Pauporte I., Romieu G., Kramar A. (2015). Cardiac toxicity events in the PHARE trial, an adjuvant trastuzumab randomised phase III study. Eur. J. Cancer.

[96] Romond E.H., Perez E.A., Bryant J., Suman V.J., Geyer C.E., Davidson N.E., Tan-Chiu E., Martino S., Paik S., Kaufman P.A. (2005). Trastuzumab plus adjuvant chemotherapy for operable HER2-positive breast cancer. N. Engl. J. Med..

[97] Slamon D., Eiermann W., Robert N., Pienkowski T., Martin M., Press M., Mackey J., Glaspy J., Chan A., Pawlicki M. (2011). Adjuvant trastuzumab in HER2-positive breast cancer. N. Engl. J. Med..

[98] Denkert C., von Minckwitz G., Darb-Esfahani S., Lederer B., Heppner B.I., Weber K.E., Budczies J., Huober J., Klauschen F., Furlanetto J. (2018). Tumour-infiltrating lymphocytes and prognosis in different subtypes of breast cancer: A pooled analysis of 3771 patients treated with neoadjuvant therapy. Lancet Oncol..

[99] Gianni L., Eiermann W., Semiglazov V., Manikhas A., Lluch A., Tjulandin S., Zambetti M., Vazquez F., Byakhow M., Lichinitser M. (2010). Neoadjuvant chemotherapy with trastuzumab followed by adjuvant trastuzumab versus neoadjuvant chemotherapy alone, in patients with HER2-positive locally advanced breast cancer (the NOAH trial): A randomised controlled superiority trial with a parallel HER2-negative cohort. Lancet.

[100] Gianni L., Pienkowski T., Im Y.-H., Tseng L.-M., Liu M.-C., Lluch A., Starosławska E., de la Haba-Rodriguez J., Im S.-A., Pedrini J.L. (2016). 5-year analysis of neoadjuvant pertuzumab and trastuzumab in patients with locally advanced, inflammatory, or early-stage HER2-positive breast cancer (NeoSphere): A multicentre, open-label, phase 2 randomised trial. Lancet Oncol..

[101] von Minckwitz G., Procter M., de Azambuja E., Zardavas D., Benyunes M., Viale G., Suter T., Arahmani A., Rouchet N., Clark E. (2017). Adjuvant Pertuzumab and Trastuzumab in Early HER2-Positive Breast Cancer. N. Engl. J. Med..

[102] Poli I., Oliva G.R., Mongelli G., Rotondi A., Frescura V., Arcuri G., Garufi G., Pontolillo L., Mastrantoni L., Di Monte E. (2026). Next Frontier in HER2+/HR+ Breast Cancer: Leveraging Cell Cycle Control with CDK4/6 Inhibitors. J. Pers. Med..

[103] Zeng Z., Zhong L., Zhu L., Liao L., Zhu Z., Cao Y., Zheng D., Huang G., Chen W., Zhang L. (2026). Subcutaneous versus intravenous trastuzumab for HER2-positive breast cancer: A global systematic review and meta-analysis with a cost-minimization analysis from the Chinese healthcare system perspective. Front. Pharmacol..

[104] von Minckwitz G., Huang C.S., Mano M.S., Loibl S., Mamounas E.P., Untch M., Wolmark N., Rastogi P., Schneeweiss A., Redondo A. (2019). Trastuzumab Emtansine for Residual Invasive HER2-Positive Breast Cancer. N. Engl. J. Med..

[105] Martín M., Pandiella A., Vargas-Castrillón E., Díaz-Rodríguez E., Iglesias-Hernangómez T., Martínez Cano C., Fernández-Cuesta I., Winkow E., Perelló M.F. (2024). Trastuzumab Deruxtecan in Breast Cancer. Crit. Rev. Oncol. Hematol..

[106] Untch M., Jackisch C., Schneeweiss A., Conrad B., Aktas B., Denkert C., Eidtmann H., Wiebringhaus H., Kümmel S., Hilfrich J. (2016). Nab-paclitaxel versus solvent-based paclitaxel in neoadjuvant chemotherapy for early breast cancer (GeparSepto-GBG 69): A randomised, phase 3 trial. Lancet Oncol..

[107] Cronin M., Kieran R., Steele C., Cooke K., O’Reilly S. (2026). Breast Cancer Patient Attitudes Towards Oncology Drug Costs in Ireland. Curr. Oncol..

[108] Shin P., Niu F., Nguyen D.D., Ho S.L., Shah N., Durna L., Hui R.L. (2026). Effectiveness and Safety of Trastuzumab-anns Compared to Reference Trastuzumab Among Patients with HER2-Positive Breast Cancer: A Non-Inferiority Study. J. Oncol. Pharm. Pract..

[109] Loibl S., Gianni L. (2017). HER2-Positive Breast Cancer. Lancet.

[110] Swain S.M., Baselga J., Kim S.-B., Ro J., Semiglazov V., Campone M., Ciruelos E., Ferrero J.-M., Schneeweiss A., Heeson S. (2015). Pertuzumab, Trastuzumab, and Docetaxel in HER2-Positive Metastatic Breast Cancer. N. Engl. J. Med..

[111] Verma S., Miles D., Gianni L., Krop I.E., Welslau M., Baselga J., Pegram M., Oh D.-Y., Diéras V., Guardino E. (2012). Trastuzumab Emtansine for HER2-Positive Advanced Breast Cancer. N. Engl. J. Med..

[112] Lee J., Park Y.H. (2022). Trastuzumab Deruxtecan for HER2+ Advanced Breast Cancer. Future Oncol..

[113] Murthy R.K., Loi S., Okines A., Paplomata E., Hamilton E., Hurvitz S.A., Lin N.U., Borges V., Abramson V., Anders C. (2020). Tucatinib, Trastuzumab, and Capecitabine for HER2-Positive Metastatic Breast Cancer. N. Engl. J. Med..

[114] Modi S., Park H., Murthy R.K., Iwata H., Tamura K., Tsurutani J., Kim S.B., Saura C., Andre F., Krop I.E. (2020). Antitumor Activity and Safety of Trastuzumab Deruxtecan in Patients With HER2-Low-Expressing Advanced Breast Cancer: Results From a Phase Ib Study. J. Clin. Oncol..

[115] Shaaban A.M., Kaur T., Provenzano E. (2025). HER2-Low Breast Cancer—Current Knowledge and Future Directions. Medicina.

[116] Xing F., Gao H., Chen G., Sun L., Sun J., Qiao X., Xue J., Liu C. (2023). CMTM6 Overexpression Confers Trastuzumab Resistance in HER2-Positive Breast Cancer. Mol. Cancer.

[117] Yang M., Li Y., Kong L., Huang S., He L., Liu P., Mo S., Lu X., Lin X., Xiao Y. (2023). Inhibition of DPAGT1 Suppresses HER2 Shedding and Trastuzumab Resistance in Human Breast Cancer. J. Clin. Investig..

[118] Zheng F., Du F., Yuan P. (2022). Trastuzumab Deruxtecan for Breast Cancer. N. Engl. J. Med..

[119] Stanowicka-Grada M., Senkus E. (2023). Anti-HER2 Drugs for the Treatment of Advanced HER2 Positive Breast Cancer. Curr. Treat. Options Oncol..

[120] Derakhshani A., Rezaei Z., Safarpour H., Sabri M., Mir A., Sanati M.A., Vahidian F., Gholamiyan Moghadam A., Aghadoukht A., Hajiasgharzadeh K. (2020). Overcoming Trastuzumab Resistance in HER2-Positive Breast Cancer Using Combination Therapy. J. Cell. Physiol..

[121] Tolaney S.M., Krop I.E. (2009). Mechanisms of Trastuzumab Resistance in Breast Cancer. Anticancer Agents Med. Chem..

[122] Schneeweiss A., Chia S., Hickish T., Harvey V., Eniu A., Hegg R., Tausch C., Seo J.H., Tsai Y.F., Ratnayake J. (2013). Pertuzumab plus Trastuzumab in Combination with Standard Neoadjuvant Anthracycline-Containing and Anthracycline-Free Chemotherapy Regimens in Patients with HER2-Positive Early Breast Cancer: A Randomized Phase II Cardiac Safety Study (TRYPHAENA). Ann. Oncol..

[123] Perez E.A., Rodeheffer R. (2004). Clinical Cardiac Tolerability of Trastuzumab. J. Clin. Oncol..

[124] Jackisch C., Kim S.-B., Semiglazov V., Melichar B., Pivot X., Hillenbach C., Stroyakovskiy D., Lum B.L., Elliott R., Weber H.A. (2015). Subcutaneous versus intravenous formulation of trastuzumab for HER2-positive early breast cancer: Updated results from the phase III HannaH study. Ann. Oncol..

[125] Heo Y.A., Syed Y.Y. (2019). Subcutaneous Trastuzumab: A Review in HER2-Positive Breast Cancer. Target Oncol..

[126] Fujiwara S., Saito N., Yasukawa M., Narita A., Sakurai-Yageta M., Sato S., Yamashita T., Hoshino D. (2026). Association Between Single-Nucleotide Polymorphism and Trastuzumab Deruxtecan-Induced Interstitial Lung Disease in Breast Cancer Using the Japonica Array NEO. Cancers.

[127] Swain S.M., Ewer M.S., Viale G., Delaloge S., Ferrero J.M., Verrill M., Colomer R., Vieira C., Werner T.L., Douthwaite H. (2013). Cardiac Tolerability of Pertuzumab plus Trastuzumab plus Docetaxel in Patients with HER2-Positive Metastatic Breast Cancer. Oncologist.

[128] Barzaman K., Karami J., Zarei Z., Hosseinzadeh A., Kazemi M.H., Moradi-Kalbolandi S., Safari E., Farahmand L. (2020). Breast Cancer: Biology, Biomarkers, and Treatments. Int. Immunopharmacol..

[129] Genentech, Inc Herceptin (Trastuzumab) Prescribing Information. https://www.gene.com/download/pdf/herceptin_prescribing.pdf.

[130] European Medicines Agency Herceptin: EPAR—Product Information. https://www.ema.europa.eu/en/medicines/human/EPAR/herceptin.

[131] Vogel C.L., Cobleigh M.A., Tripathy D., Gutheil J.C., Harris L.N., Fehrenbacher L., Slamon D.J., Murphy M., Novotny W.F., Burchmore M. (2002). Efficacy and safety of trastuzumab as a single agent in first-line treatment of HER2-overexpressing metastatic breast cancer. J. Clin. Oncol..

[132] Finn R.S., Crown J.P., Lang I., Boer K., Bondarenko I.M., Kulyk S.O., Ettl J., Patel R., Pinter T., Schmidt M. (2015). The cyclin-dependent kinase 4/6 inhibitor palbociclib in combination with letrozole versus letrozole alone as first-line treatment of oestrogen receptor-positive, HER2-negative, advanced breast cancer (PALOMA-1/TRIO-18): A randomised phase 2 study. Lancet Oncol..

[133] Spring L.M., Wander S.A., Andre F., Moy B., Turner N.C., Bardia A. (2020). Cyclin-dependent kinase 4 and 6 inhibitors for hormone receptor-positive breast cancer: Past, present, and future. Lancet.

[134] Sherr C.J., Beach D., Shapiro G.I. (2015). Targeting CDK4 and CDK6: From discovery to therapy. Cancer Discov..

[135] Fassl A., Geng Y., Sicinski P. (2022). CDK4 and CDK6 kinases: From basic science to cancer therapy. Science.

[136] Malumbres M., Barbacid M. (2009). Cell cycle, CDKs and cancer: A changing paradigm. Nat. Rev. Cancer.

[137] Patnaik A., Rosen L.S., Tolaney S.M., Tolcher A.W., Goldman J.W., Gandhi L., Papadopoulos K.P., Beeram M., Rasco D.W., Hilton J.F. (2016). Efficacy and safety of abemaciclib, an inhibitor of CDK4 and CDK6, for patients with breast cancer, non-small cell lung cancer, and other solid tumors. Cancer Discov..

[138] Kotake T., Toi M. (2018). Abemaciclib for the treatment of breast cancer. Expert Opin. Pharmacother..

[139] Goel S., DeCristo M.J., Watt A.C., BrinJones H., Sceneay J., Li B.B., Khan N., Ubellacker J.M., Xie S., Metzger-Filho O. (2017). CDK4/6 inhibition triggers anti-tumour immunity. Nature.

[140] Hurvitz S.A., Martin M., Press M.F., Chan D., Fernandez-Abad M., Petru E., Rostorfer R., Guarneri V., Huang C.S., Barriga S. (2020). Potent Cell-Cycle Inhibition and Upregulation of Immune Response with Abemaciclib and Anastrozole in neoMONARCH, Phase II Neoadjuvant Study in HR(+)/HER2(−) Breast Cancer. Clin. Cancer Res..

[141] Dickler M.N., Tolaney S.M., Rugo H.S., Cortés J., Diéras V., Patt D., Wildiers H., Hudis C.A., O’Shaughnessy J., Zamora E. (2017). MONARCH 1, a phase II study of abemaciclib, a CDK4 and CDK6 inhibitor, as a single agent, in patients with refractory HR+/HER2− metastatic breast cancer. Clin. Cancer Res..

[142] O’Shaughnessy J., Petrakova K., Sonke G.S., Conte P., Arteaga C.L., Cameron D.A., Hart L.L., Villanueva C., Jakobsen E., Beck J.T. (2018). Ribociclib plus letrozole versus letrozole alone in patients with de novo HR+, HER2− advanced breast cancer in the randomized MONALEESA-2 trial. Breast Cancer Res. Treat..

[143] Slamon D., Lipatov O., Nowecki Z., McAndrew N., Kukielka-Budny B., Stroyakovskiy D., Yardley D.A., Huang C.-S., Fasching P.A., Crown J. (2024). Ribociclib plus endocrine therapy in early breast cancer. N. Engl. J. Med..

[144] Takada M., Gunarathna S., Nguyen R., Yu M., Bonnet P., Yamada H., Nagashima T., Fujimoto H., Sakakibara J., Yamamoto H. (2026). Exploring the mechanism of action of abemaciclib in breast cancer through circulating chromatin fragments. Commun. Med..

[145] Network of Cancer Research Abemaciclib Is a Selective CDK4/6 Inhibitor for Kinds of Cancers Research. https://www.cancer-research-network.com/2024/10/09/abemaciclib-is-a-selective-cdk4-6-inhibitor-for-kinds-of-cancers-research/.

[146] Duchnowska R., Soter K., Pogoda K., Smok-Kalwat J., Grela-Wojewoda A., Winsko-Szczęsnowicz K., Kowalewska-Felczak A., Szwiec M., Danielewicz I., Streb J. (2026). Real-world outcomes with palbociclib, ribociclib, and abemaciclib plus endocrine therapy in HR+/HER2− advanced breast cancer: A multicenter retrospective study. Breast.

[147] Spring L.M., Zangardi M.L., Moy B., Bardia A. (2017). Clinical management of potential toxicities and drug interactions related to cyclin-dependent kinase 4/6 inhibitors in breast cancer: Practical considerations and recommendations. Oncologist.

[148] Sledge G.W., Toi M., Neven P., Sohn J., Inoue K., Pivot X., Burdaeva O., Okera M., Masuda N., Kaufman P.A. (2020). The Effect of Abemaciclib Plus Fulvestrant on Overall Survival in Hormone Receptor-Positive, ERBB2-Negative Breast Cancer That Progressed on Endocrine Therapy—MONARCH 2: A Randomized Clinical Trial. JAMA Oncol..

[149] Johnston S., Martin M., Di Leo A., Im S.-A., Awada A., Forrester T., Frenzel M., Hardebeck M.C., Cox J., Barriga S. (2019). MONARCH 3 final PFS: A randomized study of abemaciclib as initial therapy for advanced breast cancer. npj Breast Cancer.

[150] Goetz M.P., Toi M., Huober J., Sohn J., Trédan O., Park I.H., Campone M., Chen S.C., Manso L.M., Paluch-Shimon S. (2024). Abemaciclib plus a nonsteroidal aromatase inhibitor as initial therapy for HR+, HER2− advanced breast cancer: Final overall survival results of MONARCH 3. Ann. Oncol..

[151] Hortobagyi G.N., Stemmer S.M., Burris H.A., Yap Y.-S., Sonke G.S., Paluch-Shimon S., Campone M., Blackwell K.L., André F., Winer E.P. (2016). Ribociclib as first-line therapy for HR-positive, advanced breast cancer. N. Engl. J. Med..

[152] Cejuela M., Gil-Torralvo A., Castilla M.Á., Domínguez-Cejudo M.Á., Falcón A., Benavent M., Molina-Pinelo S., Ruiz-Borrego M., Salvador Bofill J. (2023). Abemaciclib, Palbociclib, and Ribociclib in Real-World Data: A Direct Comparison of First-Line Treatment for Endocrine-Receptor-Positive Metastatic Breast Cancer. Int. J. Mol. Sci..

[153] Brufsky A.M., Finn R.S., Metzger O., Goncalves R., Huang-Bartlett C., Sreenivasan S., Nur U., Davies J., Grigorenko A., Long G.H. (2026). Comparative effectiveness of CDK4/6 inhibitors in metastatic breast cancer: Using the target trial emulation framework to investigate overall survival in routine care. Breast Cancer Res. Treat..

[154] Blancas I., de la Peña M.G., Abad M.F., Novoa S.A., Cebrián E.A., Bayona R.S., Adelantado E.Z., Conejero R.A., Del Barco Berrón S., Atienza M. (2026). Clinical experience and satisfaction in patients with advanced breast cancer participating in the abemaciclib patient support program in Spain: A prospective observational study. Clin. Transl. Oncol..

[155] Rastogi P., O’Shaughnessy J., Martin M., Boyle F., Cortes J., Rugo H.S., Goetz M.P., Hamilton E.P., Huang C.-S., Senkus E. (2024). Adjuvant abemaciclib plus endocrine therapy for hormone receptor-positive, human epidermal growth factor receptor 2-negative, high-risk early breast cancer: Results from a preplanned monarchE overall survival interim analysis, including 5-year efficacy outcomes. J. Clin. Oncol..

[156] Harbeck N., Rastogi P., Martin M., Tolaney S.M., Shao Z.M., Fasching P.A., Huang C.S., Jaliffe G.G., Tryakin A., Goetz M.P. (2021). Adjuvant abemaciclib combined with endocrine therapy for high-risk early breast cancer: Updated efficacy and Ki-67 analysis from the monarchE study. Ann. Oncol..

[157] Rugo H.S., O’Shaughnessy J., Boyle F., Toi M., Broom R., Blancas I., Gumus M., Yamashita T., Im Y.H., Rastogi P. (2022). Adjuvant abemaciclib combined with endocrine therapy for high-risk early breast cancer: Safety and patient-reported outcomes from the monarchE study. Ann. Oncol..

[158] Tolaney S.M., Guarneri V., Seo J.H., Cruz J., Henriques Abreu M., Takahashi M., Barrios C., McIntyre K., Wei R., Munoz M. (2024). Long-term patient-reported outcomes from monarchE: Abemaciclib plus endocrine therapy as adjuvant therapy for HR+, HER2−, node-positive, high-risk, early breast cancer. Eur. J. Cancer.

[159] Obispo B., Bailleux C., Cantos B., Zamora P., Jhawar S.R., Varghese J., Cabal-Hierro L., Luz P., Berrocal-Almanza L., Xu X. (2025). Long-term adverse events following early breast cancer treatment with a focus on the BRCA-mutated population. Cancers.

[160] Freelander A., Brown L.J., Parker A., Segara D., Portman N., Lau B., Lim E. (2021). Molecular biomarkers for contemporary therapies in hormone receptor-positive breast cancer. Genes.

[161] Di Grazia G., Sánchez-Bayona R., Casals-Pascual C., Pascual T., Generali D., Gennari A., Vigneri P., Harbeck N., Cortés J., Prat A. (2026). Dynamic biomarkers in hormone receptor-positive/HER2-negative breast cancer trials: A new hope for precision oncology. npj Breast Cancer.

[162] Pauls M., Chia S. (2022). Clinical utility of genomic assay in node-positive early-stage breast cancer. Curr. Oncol..

[163] Braun M., Gluz O., Kuemmel S., Nitz U., Luedtke-Heckenkamp K., Darsow M., Forstbauer H., Polata S., Grischke E.M., Uleer C. (2026). Refining risk assessment for adjuvant CDK4/6 inhibitors beyond trial inclusion criteria: Integrating recurrence score and endocrine responsiveness according to ADAPT. Breast Care.

[164] Bartsch R., Dormann C., Egle D., Gampenrieder S.P., Pristauz-Telsnigg G., Rinnerthaler G., Singer C.F., Gnant M. (2026). Austrian treatment algorithms 2025: CDK 4/6 inhibitors in the adjuvant therapy of HR+/HER2− early breast cancer. Wien. Klin. Wochenschr..

[165] Xu Z., Zeng Y., Shen K., Hu C., Gao W. (2026). Omitting axillary lymph node dissection or not? A multicenter, retrospective study for breast cancer patients potentially eligible for abemaciclib since the SENOMAC trial. World J. Surg. Oncol..

[166] Rugo H.S., Lerebours F., Ciruelos E., Drullinsky P., Ruiz-Borrego M., Neven P., Park Y.H., Prat A., Bachelot T., Juric D. (2024). Alpelisib plus fulvestrant in PIK3CA-mutated, hormone receptor-positive advanced breast cancer after a CDK4/6 inhibitor (BYLieve): One cohort of a phase 2, multicentre, open-label, non-comparative study. Lancet Oncol..

[167] Juric D., Janku F., Rodón J., Burris H.A., Mayer I.A., Schuler M., Seggewiss-Bernhardt R., Gil-Martin M., Middleton M.R., Baselga J. (2019). Alpelisib plus fulvestrant in PIK3CA-altered and PIK3CA-wild-type estrogen receptor-positive advanced breast cancer: A phase 1b clinical trial. JAMA Oncol..

[168] Papadimitriou M.C., Pazaiti A., Iliakopoulos K., Markouli M., Michalaki V., Papadimitriou C.A. (2022). Resistance to CDK4/6 inhibition: Mechanisms and strategies to overcome a therapeutic problem in the treatment of hormone receptor-positive metastatic breast cancer. Biochim. Biophys. Acta Mol. Cell Res..

[169] Herrera-Abreu M.T., Palafox M., Asghar U., Rivas M.A., Cutts R.J., Garcia-Murillas I., Pearson A., Guzman M., Rodriguez O., Grueso J. (2016). Early adaptation and acquired resistance to CDK4/6 inhibition in estrogen receptor-positive breast cancer. Cancer Res..

[170] Kalinsky K., Bianchini G., Hamilton E., Graff S.L., Park K.H., Jeselsohn R., Demirci U., Martin M., Layman R.M., Hurvitz S.A. (2025). Abemaciclib Plus Fulvestrant in Advanced Breast Cancer After Progression on CDK4/6 Inhibition: Results From the Phase III postMONARCH Trial. J. Clin. Oncol..

[171] Tolaney S.M., Wardley A.M., Zambelli S., Hilton J.F., Troso-Sandoval T.A., Ricci F., Im S.A., Kim S.B., Johnston S.R., Chan A. (2020). Abemaciclib plus trastuzumab with or without fulvestrant versus trastuzumab plus standard-of-care chemotherapy in women with hormone receptor-positive, HER2-positive advanced breast cancer (monarcHER): A randomised, open-label, phase 2 trial. Lancet Oncol..

[172] Jhaveri K., Bidard F.C., Kalinsky K., Neven P., Rugo H.S., Tolaney S.M., Litchfield L.M., von Laue C.C., Traore S., Sapunar F. (2026). Imlunestrant plus abemaciclib versus fulvestrant plus abemaciclib in ER-positive, HER2-negative advanced breast cancer: An indirect treatment comparison of three phase III trials. ESMO Open.

[173] Dent S., Cortés J., Im Y.-H., Diéras V., Harbeck N., Krop I.E., Wilson T.R., Cui N., Schimmoller F., Hsu J.Y. (2020). Phase III randomized study of taselisib or placebo with fulvestrant in estrogen receptor-positive, PIK3CA-mutant, HER2-negative, advanced breast cancer: The SANDPIPER trial. Ann. Oncol..

[174] Baselga J., Im S.-A., Iwata H., Cortés J., De Laurentiis M., Jiang Z., Arteaga C.L., Jonat W., Clemons M., Ito Y. (2017). Buparlisib plus fulvestrant versus placebo plus fulvestrant in postmenopausal, hormone receptor-positive, HER2-negative, advanced breast cancer (BELLE-2): A randomised, double-blind, placebo-controlled, phase 3 trial. Lancet Oncol..

[175] Di Leo A., Johnston S., Lee K.S., Ciruelos E., Lønning P.E., Janni W., O’Regan R., Mouret-Reynier M.-A., Kalev D., Egle D. (2018). Buparlisib plus fulvestrant in postmenopausal women with hormone-receptor-positive, HER2-negative, advanced breast cancer progressing on or after mTOR inhibition (BELLE-3): A randomised, double-blind, placebo-controlled, phase 3 trial. Lancet Oncol..

[176] Jhaveri K.L., Neven P., Casalnuovo M.L., Kim S.B., Tokunaga E., Aftimos P., Saura C., O’Shaughnessy J., Harbeck N., Carey L.A. (2025). Imlunestrant with or without Abemaciclib in Advanced Breast Cancer. N. Engl. J. Med..

[177] Jhaveri K.L., Neven P., Casalnuovo M.L., Kim S.B., Tokunaga E., Aftimos P., Saura C., O’Shaughnessy J., Harbeck N., Carey L.A. (2026). Imlunestrant with or without abemaciclib in advanced breast cancer: Updated efficacy results from the phase III EMBER-3 trial. Ann. Oncol..

[178] Hua M., Xiong F., Chong S., Zhang Z., Liu Q., Hou J., Zhang Z., Gu Z., Cui X., Cui Y. (2024). Abemaciclib increases the risk of venous thromboembolism in breast cancer: Integrated meta-analysis, pharmacovigilance database analysis, and in vitro validation. Cancer Treat. Rev..

[179] Alsuhebany N., Alanazi A., Alzahrani M.Y., Alyousef S., Aldawish B.S., Aljardan S., Abu Alnasr R., Althumali I.M., AlDoughaim M., Alfehaid L. (2026). Assessing thrombotic risk in patients with HR+/HER2− breast cancer receiving CDK4/6 inhibitors: Insights from real-world data from the Middle East. Breast Cancer.

[180] Davis N.E., Herrmann J., Hodge D.O., Blumenfeld S., Ruddy K.J., Tan N.Y. (2026). Incidence and outcomes of atrial arrhythmia with cyclin dependent kinase 4/6 inhibitors in hormone receptor-positive/human epidermal growth factor receptor 2-negative breast cancer. Int. J. Cardiol..

[181] Xie M., Yang F., Zhu S., Xu Y., Li X. (2026). Post-marketing safety surveillance and adverse event risk factors of abemaciclib: A pharmacoepidemiologic study. Naunyn Schmiedebergs Arch. Pharmacol..

[182] Finn R.S., Rugo H.S., Gelmon K.A., Cristofanilli M., Colleoni M., Loi S., Schnell P., Lu D.R., Puyana Theall K., Mori A. (2021). Long-term pooled safety analysis of palbociclib in combination with endocrine therapy for hormone receptor-positive/human epidermal growth factor receptor 2-negative advanced breast cancer: Updated analysis with up to 5 years of follow-up. Oncologist.

[183] Fisusi F.A., Akala E.O. (2019). Drug combinations in breast cancer therapy. Pharm. Nanotechnol..

[184] Sisodiya S., Kasherwal V., Rani J., Mishra N., Kumar S., Khan A., Aftab M., Shagufta, Singh P., Gupta E. (2024). Impact of combinatorial immunotherapies in breast cancer: A systematic review and meta-analysis. Front. Immunol..

[185] Lee J.S., Yost S.E., Li S.M., Cui Y., Frankel P.H., Yuan Y.-C., Schmolze D., Egelston C.A., Guo W., Murga M. (2022). Genomic markers of CDK 4/6 inhibitor resistance in hormone receptor positive metastatic breast cancer. Cancers.

[186] Ogata R., Kishino E., Saitoh W., Koike Y., Kurebayashi J. (2021). Resistance to cyclin-dependent kinase (CDK) 4/6 inhibitors confers cross-resistance to other CDK inhibitors but not to chemotherapeutic agents in breast cancer cells. Breast Cancer.

[187] Villarreal-Garza C., Meraz-Brenez A., Reyes Morales A., Mushtaq A.W., Moreno-Jaime B., Landaverde D.U., Petracci F.E., Idrobo Quintero H., Moreno Ríos J., Pérez-Vargas J.C.S. (2026). Preferences in Cyclin-Dependent Kinase 4/6 Inhibitors for Advanced Breast Cancer Among Medical Oncologists in Latin America. JCO Glob. Oncol..

[188] Sandeep K.S., Appachu S., Diwaker R.B., Vivek S., Simha V.V.S. (2025). Clinical outcomes and safety study of CDK4/6 inhibitors in hormone-positive metastatic breast cancer—A real-world tertiary cancer center experience. Indian J. Cancer.

[189] Cristofanilli M., Turner N.C., Bondarenko I., Ro J., Im S.-A., Masuda N., Colleoni M., DeMichele A., Loi S., Verma S. (2016). Fulvestrant plus palbociclib versus fulvestrant plus placebo for treatment of hormone-receptor-positive, HER2-negative metastatic breast cancer that progressed on previous endocrine therapy (PALOMA-3): Final analysis of the multicentre, double-blind, phase 3 randomised controlled trial. Lancet Oncol..

[190] Ismaili N. (2026). A systematic review of major advances in breast cancer therapeutics in 2025: Synthesis of conference and published evidence. Int. J. Mol. Sci..

[191] Eli Lilly and Company Verzenio (Abemaciclib) Prescribing Information. https://pi.lilly.com/us/verzenio-uspi.pdf.

[192] European Medicines Agency Verzenio: EPAR—Product Information. https://www.ema.europa.eu/en/medicines/human/EPAR/verzenios.

[193] Zhang Q.Y., Sun T., Yin Y.M., Li H.P., Yan M., Tong Z.S., Oppermann C.P., Liu Y.P., Costa R., Li M. (2020). MONARCH plus: Abemaciclib plus endocrine therapy in women with HR+/HER2- advanced breast cancer: The multinational randomized phase III study. Ther. Adv. Med. Oncol..

[194] Johnston S.R.D., Harbeck N., Hegg R., Toi M., Martin M., Shao Z.M., Zhang Q.Y., Martinez Rodriguez J.L., Campone M., Hamilton E. (2020). Abemaciclib combined with endocrine therapy for the adjuvant treatment of HR+, HER2-, node-positive, high-risk, early breast cancer (monarchE). J. Clin. Oncol..

[195] Hamilton E., Cortés J., Ozyilkan O., Chen S.-C., Petrakova K., Manikhas A., Jerusalem G., Hegg R., Huober J., Zhang W. (2022). nextMONARCH phase 2 randomized clinical trial: Overall survival analysis of abemaciclib monotherapy or in combination with tamoxifen in patients with endocrine-refractory HR+, HER2- metastatic breast cancer. Breast Cancer Res. Treat..

[196] Samuels Y., Waldman T. (2010). Oncogenic mutations of PIK3CA in human cancers. Curr. Top. Microbiol. Immunol..

[197] Martínez-Sáez O., Chic N., Pascual T., Adamo B., Vidal M., González-Farré B., Sanfeliu E., Schettini F., Conte B., Brasó-Maristany F. (2020). Frequency and spectrum of PIK3CA somatic mutations in breast cancer. Breast Cancer Res..

[198] Mavratzas A., Marmé F. (2021). Alpelisib in the treatment of metastatic HR+ breast cancer with PIK3CA mutations. Future Oncol..

[199] Fruman D.A., Rommel C. (2014). PI3K and cancer: Lessons, challenges and opportunities. Nat. Rev. Drug Discov..

[200] Copur M.S. (2020). Alpelisib to treat breast cancer. Drugs Today.

[201] Chandak S.M., Kumbhare M.R., Patole V.P. (2025). Unlocking the potential of alpelisib in breast cancer: A comprehensive review. Chin. J. Appl. Physiol..

[202] Miller T.W., Balko J.M., Arteaga C.L. (2011). Phosphatidylinositol 3-kinase and antiestrogen resistance in breast cancer. J. Clin. Oncol..

[203] Alves C.L., Ditzel H.J. (2023). Drugging the PI3K/AKT/mTOR pathway in ER+ breast cancer. Int. J. Mol. Sci..

[204] Hao C., Wei Y., Meng W., Zhang J., Yang X. (2025). PI3K/AKT/mTOR inhibitors for hormone receptor-positive advanced breast cancer. Cancer Treat. Rev..

[205] Baselga J. (2011). Targeting the phosphoinositide-3 (PI3) kinase pathway in breast cancer. Oncologist.

[206] Porta C., Paglino C., Mosca A. (2014). Targeting PI3K/Akt/mTOR signaling in cancer. Front. Oncol..

[207] Browne I.M., André F., Chandarlapaty S., Carey L.A., Turner N.C. (2024). Optimal targeting of PI3K–AKT and mTOR in advanced oestrogen receptor-positive breast cancer. Lancet Oncol..

[208] Turner N.C., Neven P., Loibl S., André F. (2017). Advances in the treatment of advanced oestrogen-receptor-positive breast cancer. Lancet.

[209] Armaghani A.J., Han H.S. (2020). Alpelisib in the treatment of breast cancer: A short review on the emerging clinical data. Breast Cancer.

[210] Alves C.L., Karimi L., Terp M.G., Jakobsen M.K., Zhou F.H., Policastro B., Nissen N., Johansen L.E., Ravnsborg T., Eshraghi L. (2025). Dual PI3K/mTOR inhibition is required to combat resistance to CDK4/6 inhibitor and endocrine therapy in PIK3CA-mutant breast cancer. Sci. Transl. Med..

[211] Network of Cancer Research Alpelisib Is an Orally Active PI3Kα Inhibitor for Breast Cancer Research. https://www.cancer-research-network.com/2024/09/03/alpelisib-is-an-orally-active-pi3k%CE%B1-inhibitor-for-breast-cancer-research/.

[212] André F., Ciruelos E.M., Juric D., Loibl S., Campone M., Mayer I.A., Rubovszky G., Yamashita T., Kaufman B., Lu Y.-S. (2021). Alpelisib plus fulvestrant for PIK3CA-mutated, hormone receptor-positive, human epidermal growth factor receptor-2-negative advanced breast cancer: Final overall survival results from SOLAR-1. Ann. Oncol..

[213] Cardoso F., Paluch-Shimon S., Senkus E., Curigliano G., Aapro M.S., André F., Barrios C.H., Bergh J., Bhattacharyya G.S., Biganzoli L. (2020). 5th ESO-ESMO international consensus guidelines for advanced breast cancer (ABC 5). Ann. Oncol..

[214] Alshehri A., Altwairgi A.K., AlTourah A., Alwbari A., Chehal A., Calaud F., Al-Hashem H., Marashi H., Elsamany S., Tirmazy S.H. (2026). Optimizing second-line endocrine-based treatment in HR-positive HER2-negative metastatic breast cancer: A comprehensive expert statement from the Gulf Cooperation Council region. Front. Oncol..

[215] Mayer I.A., Abramson V.G., Formisano L., Balko J.M., Estrada M.V., Sanders M.E., Juric D., Solit D., Berger M.F., Won H.H. (2017). A Phase Ib Study of Alpelisib (BYL719), a PI3Kα-Specific Inhibitor, with Letrozole in ER+/HER2- Metastatic Breast Cancer. Clin. Cancer Res..

[216] Rugo H.S., André F., Yamashita T., Cerda H., Toledano I., Stemmer S.M., Jurado J.C., Juric D., Mayer I., Ciruelos E.M. (2020). Time course and management of key adverse events during the randomized phase III SOLAR-1 study of PI3K inhibitor alpelisib plus fulvestrant in patients with HR-positive advanced breast cancer. Ann. Oncol..

[217] Shen S., Teysir J., Safonov A., Bromberg M., Chen Y., Ahmed M., Razavi P., Jhaveri K. (2026). Sequential use of PI3K/AKT/mTOR pathway inhibitors alpelisib and everolimus in patients with hormone receptor-positive metastatic breast cancer. Breast Cancer Res..

[218] Jhaveri K.L., Iyengar N.M., Turner N.C., Rugo H.S., O’Shaughnessy J., Barrios C.H., Curigliano G., André F., Im S.-A., Goncalves M.D. (2026). Clinical management of common toxicities with inhibitors targeting the PI3K/AKT/mTOR pathway in breast cancer. ESMO Open.

[219] Loizidis S., Michaelides D., Marcou Y., Kakouri E., Konstantinou I., Constantinidou A., Theophanous-Kitiri S., Papageorgiou E., Michailidou K., Galazi M. (2026). Comparison between alpelisib plus endocrine therapy and everolimus plus endocrine therapy after CDK4/6 inhibitor progression in patients with PIK3CA-mutant metastatic breast cancer: A single-center retrospective study. Cancers.

[220] Rodon J., Dienstmann R., Serra V., Tabernero J. (2013). Development of PI3K inhibitors: Lessons learned from early clinical trials. Nat. Rev. Clin. Oncol..

[221] Harbeck N., Gnant M. (2017). Breast cancer. Lancet.

[222] Peixe C., Vicente Rocha J., Inês Alexandre M. (2026). Severe hyperglycemia induced by alpelisib in breast cancer: A call for early monitoring. Med. Clin..

[223] Marni M., Simoens D., Romero N., Jones W.K., Kaja S. (2025). Disproportionality analysis of oral toxicities associated with PI3K/AKT/mTOR pathway inhibitors using the FAERS database. Pharmaceuticals.

[224] Liu Y., Hu Y., Xue J., Li J., Yi J., Bu J., Zhang Z., Qiu P., Gu X. (2023). Advances in immunotherapy for triple-negative breast cancer. Mol. Cancer.

[225] Wu W., He Y., Zhang C. (2026). Emerging targeted and multimodal therapeutic strategies in breast cancer: A comprehensive review. Breast Cancer.

[226] Annoor A., Rahman Marzan M., Iqbal R.B., Ferdausi A., Yasmeen A., Tarannum P., John P. (2025). Alpelisib-induced hyperglycemia in PIK3CA(+) breast cancer patients. South. Med. J..

[227] Yap T.A., Bjerke L., Clarke P.A., Workman P. (2015). Drugging PI3K in cancer: Refining targets and therapeutic strategies. Curr. Opin. Pharmacol..

[228] Janku F., Yap T.A., Meric-Bernstam F. (2018). Targeting the PI3K pathway in cancer: Are we making headway?. Nat. Rev. Clin. Oncol..

[229] Vasan N., Toska E., Scaltriti M. (2019). Overview of the relevance of PI3K pathway in HR-positive breast cancer. Ann. Oncol..

[230] Gough S.M., Flanagan J.J., Teh J., Andreoli M., Rousseau E., Pannone M., Bookbinder M., Willard R., Davenport K., Bortolon E. (2024). Oral estrogen receptor PROTAC vepdegestrant (ARV-471) is highly efficacious as monotherapy and in combination with CDK4/6 or PI3K/mTOR pathway inhibitors in preclinical ER+ breast cancer models. Clin. Cancer Res..

[231] Jhaveri K.L., Lim E., Jeselsohn R., Ma C.X., Hamilton E.P., Osborne C., Bhave M., Kaufman P.A., Beck J.T., Manso Sanchez L. (2024). Imlunestrant, an oral selective estrogen receptor degrader, as monotherapy and in combination with targeted therapy in estrogen receptor-positive, human epidermal growth factor receptor 2-negative advanced breast cancer: Phase Ia/Ib EMBER study. J. Clin. Oncol..

[232] Turner N.C., Liu Y., Zhu Z., Loi S., Colleoni M., Loibl S., DeMichele A., Harbeck N., André F., Bayar M.A. (2019). Cyclin E1 Expression and Palbociclib Efficacy in Previously Treated Hormone Receptor-Positive Metastatic Breast Cancer. J. Clin. Oncol..

[233] Mosele F., Stefanovska B., Lusque A., Tran Dien A., Garberis I., Droin N., Le Tourneau C., Sablin M.-P., Lacroix L., Enrico D. (2020). Outcome and molecular landscape of patients with PIK3CA-mutated metastatic breast cancer. Ann. Oncol..

[234] Zhu K., Wu Y., He P., Fan Y., Zhong X., Zheng H., Luo T. (2022). PI3K/AKT/mTOR-Targeted Therapy for Breast Cancer. Cells.

[235] Lee Y., Ni J., Beretov J., Wasinger V.C., Graham P., Li Y. (2023). Recent advances of small extracellular vesicle biomarkers in breast cancer diagnosis and prognosis. Mol. Cancer.

[236] Shi Y., Niu P., Cheng Q., Chen L., Weng Y., Yang X. (2025). Metformin enhances alpelisib sensitivity in HER2+ breast cancer by suppressing cancer stemness and oncogenic signaling. Front. Oncol..

[237] Hou Y., Qin H., Zheng X., Zhu Y., Wang X., Wang L., Min W., Cao S., Yang P. (2025). Structure-guided design of a highly selective PI3Kα inhibitor overcoming metabolic dysregulation with potent anti-breast cancer efficacy. J. Med. Chem..

[238] Weigelt B., Downward J. (2012). Genomic determinants of PI3K pathway inhibitor response in cancer. Front. Oncol..

[239] Hanker A.B., Sudhan D.R., Arteaga C.L. (2020). Overcoming endocrine resistance in breast cancer. Cancer Cell.

[240] Pontolillo L., Davis A.A., Gerratana L., Medford A.J., Wang J., Nicolo’ E., Clifton K., Velimirovic M., Warrior S., Podany E. (2025). Circulating genomic landscape following cyclin-dependent kinase 4/6 inhibitors exposure in HR+/HER2- metastatic breast cancer: A retrospective multi-institutional Consortium analysis. npj Breast Cancer.

[241] Juric D., Castel P., Griffith M., Griffith O.L., Won H.H., Ellis H., Ebbesen S.H., Ainscough B.J., Ramu A., Iyer G. (2015). Convergent loss of PTEN leads to clinical resistance to a PI(3)Kα inhibitor. Nature.

[242] Boonma T., Nutho B., Kanjanasirirat P., Rajchakom C., Nunthaboot N. (2026). Discovery of a novel PI3Kα inhibitor for breast cancer therapy via virtual screening method, molecular dynamics simulation and biological evaluation. J. Mol. Graph. Model..

[243] Novartis Pharmaceuticals Corporation Piqray (Alpelisib) Prescribing Information. https://www.novartis.com/us-en/sites/novartis_us/files/piqray.pdf.

[244] European Medicines Agency Piqray: EPAR—Product Information. https://www.ema.europa.eu/en/medicines/human/EPAR/piqray.

[245] Campone M., Im S.-A., Iwata H., Clemons M., Ito Y., Awada A., Chia S., Jagiełło-Gruszfeld A., Pistilli B., Tseng L.-M. (2018). Buparlisib plus fulvestrant versus placebo plus fulvestrant for postmenopausal, hormone receptor-positive, human epidermal growth factor receptor 2-negative, advanced breast cancer: Overall survival results from BELLE-2. Eur. J. Cancer.

[246] Cardoso F., Paluch-Shimon S., Schumacher-Wulf E., Matos L., Gelmon K., Aapro M.S., Bajpai J., Barrios C.H., Bergh J., Bergsten-Nordström E. (2024). 6th and 7th International consensus guidelines for the management of advanced breast cancer (ABC guidelines 6 and 7). Breast.

[247] Harbeck N., Penault-Llorca F., Cortes J., Gnant M., Houssami N., Poortmans P., Ruddy K., Tsang J., Cardoso F. (2019). Breast cancer. Nat. Rev. Dis. Primers.

[248] Loibl S., Poortmans P., Morrow M., Denkert C., Curigliano G. (2021). Breast cancer. Lancet.

[249] Demir Cetinkaya B., Biray Avci C. (2022). Molecular perspective on targeted therapy in breast cancer: A review of current status. Med. Oncol..

[250] Lin T.S., Wan J., He J., Cui S., Huang Y., Zhang B., Huang H.Y., Zhu K., Chen J., Zhang T. (2026). Arecoline as a Novel Scaffold Targeting the ATAD2 Bromodomain for Cell Cycle Modulation. Pharmaceutics.

[251] Fisher B., Anderson S., Bryant J., Margolese R.G., Deutsch M., Fisher E.R., Jeong J.-H., Wolmark N. (2002). Twenty-year follow-up of a randomized trial comparing total mastectomy, lumpectomy, and lumpectomy plus irradiation for the treatment of invasive breast cancer. N. Engl. J. Med..

[252] Darby S., McGale P., Correa C., Taylor C., Arriagada R., Clarke M., Cutter D., Davies C., Ewertz M., Godwin J. (2011). Effect of radiotherapy after breast-conserving surgery on 10-year recurrence and 15-year breast cancer death: Meta-analysis of individual patient data for 10,801 women in 17 randomised trials. Lancet.

[253] Burstein H.J., Curigliano G., Thürlimann B., Weber W.P., Poortmans P., Regan M.M., Senn H.J., Winer E.P. (2021). Customizing local and systemic therapies for women with early breast cancer: The St. Gallen International Consensus Guidelines for treatment of early breast cancer 2021. Ann. Oncol..

[254] Baum M., Budzar A.U., Cuzick J., Forbes J., Houghton J.H., Klijn J.G.M., Sahmoud T. (2002). Anastrozole alone or in combination with tamoxifen versus tamoxifen alone for adjuvant treatment of postmenopausal women with early breast cancer: First results of the ATAC randomised trial. Lancet.

[255] Thürlimann B., Keshaviah A., Coates A.S., Mouridsen H., Mauriac L., Forbes J.F., Paridaens R., Castiglione-Gertsch M., Gelber R.D., Rabaglio M. (2005). A comparison of letrozole and tamoxifen in postmenopausal women with early breast cancer. N. Engl. J. Med..

[256] Turner N.C., Slamon D.J., Ro J., Bondarenko I., Im S.-A., Masuda N., Colleoni M., DeMichele A., Loi S., Verma S. (2018). Overall survival with palbociclib and fulvestrant in advanced breast cancer. N. Engl. J. Med..

[257] Hortobagyi G.N., Stemmer S.M., Burris H.A., Yap Y.S., Sonke G.S., Paluch-Shimon S., Campone M., Petrakova K., Blackwell K.L., Winer E.P. (2018). Updated results from MONALEESA-2, a phase III trial of first-line ribociclib plus letrozole versus placebo plus letrozole in hormone receptor-positive, HER2-negative advanced breast cancer. Ann. Oncol..

[258] Sturzu A., Ma R., Xi Y. (2026). Circulating MicroRNA in Breast Cancer. Cancers.

[259] Bardia A., Cortés J., Bidard F.-C., Neven P., Garcia-Sáenz J., Aftimos P., O’Shaughnessy J., Lu J., Tonini G., Scartoni S. (2024). Elacestrant in ER+, HER2− metastatic breast cancer with ESR1-mutated tumors: Subgroup analyses from the phase III EMERALD trial by prior duration of endocrine therapy plus CDK4/6 inhibitor and in clinical subgroups. Clin. Cancer Res..

[260] Agostinetto E., Curigliano G., Piccart M. (2024). Emerging treatments in HER2-positive advanced breast cancer: Keep raising the bar. Cell Rep. Med..

[261] Holohan C., Van Schaeybroeck S., Longley D.B., Johnston P.G. (2013). Cancer drug resistance: An evolving paradigm. Nat. Rev. Cancer.

[262] Turner N.C., Oliveira M., Howell S.J., Dalenc F., Cortes J., Gomez Moreno H.L., Hu X., Jhaveri K., Krivorotko P., Loibl S. (2023). Capivasertib in hormone receptor-positive advanced breast cancer. N. Engl. J. Med..

[263] Krasniqi E., Goeman F., Pulito C., Palcau A.C., Ciuffreda L., Di Lisa F.S., Filomeno L., Barba M., Pizzuti L., Cappuzzo F. (2022). Biomarkers of response and resistance to CDK4/6 inhibitors in breast cancer: Hints from liquid biopsy and microRNA exploration. Int. J. Mol. Sci..

[264] Xu L., Kong X., Zhang B., Ma H., Li X., Deng Y., Liu W., Ren W., Tang X., Zhang D. (2026). Interplay Between Poly(ADP-ribosyl)ation and Specific Inner Cellular Events That Suggest Combination Strategies for Overcoming PARP Inhibitor Resistance. Pharmaceutics.

[265] Huang L., Yang Y., Duan D., Dai L., Zhai B., Qian B. (2026). Epacadostat and Olaparib Synergistically Inhibit the Growth of BRCA-Proficient Triple-Negative Breast Cancer by Suppressing the Expression of BRCA1 and RAD51. Molecules.

[266] Cortazar P., Zhang L., Untch M., Mehta K., Costantino J.P., Wolmark N., Bonnefoi H., Cameron D., Gianni L., Valagussa P. (2014). Pathological complete response and long-term clinical benefit in breast cancer: The CTNeoBC pooled analysis. Lancet.

[267] Schmid P., Cortes J., Pusztai L., McArthur H., Kümmel S., Bergh J., Denkert C., Park Y.H., Hui R., Harbeck N. (2020). Pembrolizumab for early triple-negative breast cancer. N. Engl. J. Med..

[268] Tsagkaraki I., Gannon I., Rampotas A., Singh D., Roddy H., Ottaviani D., Roddie C. (2026). Immunotherapies for Breast Cancer: From Checkpoint Inhibition to Emerging Cellular Therapies. Cancers.

[269] Leon-Ferre R.A., Goetz M.P. (2023). Advances in systemic therapies for triple negative breast cancer. BMJ.

[270] Akbari B., Hasan M.M., Islam S.M. (2025). Advances in targeted therapy for triple-negative breast cancer: A review of key antigens and recent advances. J. Drug Target..

[271] Deepak K.G.K., Vempati R., Nagaraju G.P., Dasari V.R., S N., Rao D.N., Malla R.R. (2020). Tumor microenvironment: Challenges and opportunities in targeting metastasis of triple negative breast cancer. Pharmacol. Res..

[272] Litton J.K., Rugo H.S., Ettl J., Hurvitz S.A., Gonçalves A., Lee K.-H., Fehrenbacher L., Yerushalmi R., Mina L.A., Martin M. (2018). Talazoparib in patients with advanced breast cancer and a germline BRCA mutation. N. Engl. J. Med..

[273] Bardia A., Hurvitz S.A., Tolaney S.M., Loirat D., Punie K., Oliveira M., Brufsky A., Sardesai S.D., Kalinsky K., Zelnak A.B. (2021). Sacituzumab govitecan in metastatic triple-negative breast cancer. N. Engl. J. Med..

[274] Coleman R., Hadji P., Body J.-J., Santini D., Chow E., Terpos E., Oudard S., Bruland Ø., Flamen P., Kurth A. (2020). Bone health in cancer: ESMO Clinical Practice Guidelines. Ann. Oncol..

[275] Arjsri P., Semmarath W., Srisawad K., Intanil I., Thippraphan P., Dejkriengkraikul P. (2026). Pectolinarigenin from Tiliacora triandra Exhibits Potent Anticancer Activity in Triple-Negative Breast Cancer Cells through Cell Cycle Arrest, Apoptosis, and MAPK Signaling Inhibition. Pharmaceuticals.

[276] He Q., Luo L., Zhang D., Zhou W., Bai N., Du C., Li S. (2026). Molecular Mechanisms Underlying the Anti-Tumor Activity of Lotus-Derived Alkaloids in Breast Cancer. Molecules.

[277] Gülüm L., Güler E., Çapkınoğlu E., Çelik A.B., Tutar Y. (2026). From Phytochemical Characterization to Energy Metabolism-Driven Molecular Responses: The Anticancer Potential of *Lactarius deliciosus* (L.) Gray in Breast Cancer Cells. Nutrients.

[278] Crintea A., Bocșan C.I., Jianu E.M., Șovrea A.S., Munteanu C., Kubelac M.P., Crăciun A.M., Silaghi C.N. (2026). Overcoming Chemotherapy Resistance in Triple-Negative Breast Cancer with Nanocarrier-Delivered siRNA Therapeutics. J. Clin. Med..

[279] Mukherjee D., Raikwar S. (2024). Recent Update on Nanocarrier(s) as the Targeted Therapy for Breast Cancer. AAPS PharmSciTech.

[280] Smith S., Kim A., Sony A., Aslam M., Torruella E., de la Parra C., Sauane M. (2026). Inhibitory Effect of Interleukin-24 on Programmed Death Ligand 1 Expression via a Eukaryotic Translation Initiation Factor 2 Alpha Kinase 2-Dependent Pathway in Human Triple-Negative Breast Cancer. Genes.

[281] Dogan Turacli I., Cingir Koker S., Paspal Eroglu K., Yalcin B. (2026). Targeting Activated Pathways in Doxorubicin-Resistant TNBC Alters Signaling, Survival and EMT: A Double-Edged Sword. Int. J. Mol. Sci..

[282] Chowaniec H., Ślubowska A., Mroczek M., Borowczyk M., Braszka M., Dworacki G., Dobosz P., Wichtowski M. (2024). New hopes for the breast cancer treatment: Perspectives on the oncolytic virus therapy. Front. Immunol..

[283] Zhang B., Shi H., Wang H. (2023). Machine learning and AI in cancer prognosis, prediction, and treatment selection: A critical approach. J. Multidiscip. Healthc..

[284] Lee E.Y., Lee D.-W., Lee K.-H., Im S.-A. (2023). Recent developments in the therapeutic landscape of advanced or metastatic hormone receptor–positive breast cancer. Cancer Res. Treat..

[285] Bruxelles Q., Hamel-Côté G., Scott-Boyer M.P., Ouellette V., C-Gaudreault R., Durocher F., Diorio C., Droit A., Fortin S. (2026). FOXA1 and RAB25 as Biomarkers of Breast Cancer Cell Response to CYP1A1-Activated Prodrugs: Insights from CEU-938. Pharmaceuticals.

[286] Wolff A.C., Hammond M.E.H., Allison K.H., Harvey B.E., Mangu P.B., Bartlett J.M.S., Bilous M., Ellis I.O., Fitzgibbons P., Hanna W. (2018). Human Epidermal Growth Factor Receptor 2 Testing in Breast Cancer: American Society of Clinical Oncology/College of American Pathologists Clinical Practice Guideline Focused Update. J. Clin. Oncol..

[287] Allison K.H., Hammond M.E.H., Dowsett M., McKernin S.E., Carey L.A., Fitzgibbons P.L., Hayes D.F., Lakhani S.R., Chavez-MacGregor M., Perlmutter J. (2020). Estrogen and Progesterone Receptor Testing in Breast Cancer: ASCO/CAP Guideline Update. J. Clin. Oncol..

[288] Compton C.C., Robb J.A., Anderson M.W., Berry A.B., Birdsong G.G., Bloom K.J., Branton P.A., Crothers J.W., Cushman-Vokoun A.M., Hicks D.G. (2019). Preanalytics and Precision Pathology: Pathology Practices to Ensure Molecular Integrity of Cancer Patient Biospecimens for Precision Medicine. Arch. Pathol. Lab. Med..

[289] Curtit E., Nerich V., Mansi L., Chaigneau L., Cals L., Villanueva C., Bazan F., Montcuquet P., Meneveau N., Perrin S. (2013). Discordances in Estrogen Receptor Status, Progesterone Receptor Status, and HER2 Status Between Primary Breast Cancer and Metastasis. Oncologist.

[290] Shanthala S., Amirtham U., Gopal C., M.N. S., Jacob L., Babu G. (2023). Study of Biomarker Discordance between Primary and Recurrent Sites and Its Clinical Implications in Metastatic Breast Cancer: A Single Institutional Study from India. South Asian J. Cancer.

[291] Ross J.S., Ali S.M., Wang K., Khaira D., Palma N.A., Chmielecki J., Palmer G.A., Morosini D., Elvin J.A., Fernandez S.V. (2015). Comprehensive Genomic Profiling of Inflammatory Breast Cancer Cases Reveals a High Frequency of Clinically Relevant Genomic Alterations. Breast Cancer Res. Treat..

[292] Cristofanilli M., Rugo H.S., Im S.-A., Slamon D.J., Harbeck N., Bondarenko I., Masuda N., Colleoni M., DeMichele A., Loi S. (2022). Overall survival with palbociclib and fulvestrant in women with HR+/HER2- ABC: Updated exploratory analyses of PALOMA-3, a double-blind, phase III randomized study. Clin. Cancer Res..

[293] Peto R., Davies C., Godwin J., Gray R., Pan H.C., Clarke M., Cutter D., Darby S., McGale P., Early Breast Cancer Trialists’ Collaborative Group (EBCTCG) (2012). Comparisons between different polychemotherapy regimens for early breast cancer: Meta-analyses of long-term outcome among 100,000 women in 123 randomised trials. Lancet.

[294] Cortés J., Rugo H.S., Cescon D.W., Im S.-A., Yusof M.M., Gallardo C., Lipatov O., Barrios C.H., Perez-Garcia J., Iwata H. (2022). Pembrolizumab plus chemotherapy in advanced triple-negative breast cancer. N. Engl. J. Med..

[295] Modi S., Jacot W., Iwata H., Park Y.H., Vidal Losada M., Li W., Tsurutani J., Ueno N.T., Zaman K., Prat A. (2025). Trastuzumab deruxtecan in HER2-low metastatic breast cancer: Long-term survival analysis of the randomized, phase 3 DESTINY-Breast04 trial. Nat. Med..

[296] Schiavon G., Hrebien S., Garcia-Murillas I., Cutts R.J., Pearson A., Tarazona N., Fenwick K., Kozarewa I., Lopez-Knowles E., Ribas R. (2015). Analysis of ESR1 mutation in circulating tumor DNA demonstrates evolution during therapy for metastatic breast cancer. Sci. Transl. Med..

